# Cell-surface photochemistry mediated calcium overload for synergistic tumor therapy

**DOI:** 10.1186/s12951-023-02090-z

**Published:** 2023-09-19

**Authors:** Jun Wang, Wei Wang, Qingmei Shen, Lan Lan, Cuiping Guan, Xinchang Xu, Weishuo Li, Yongzhong Du

**Affiliations:** 1 Department of Pharmacy, Hangzhou Third People’s Hospital, Affiliated Hangzhou Dermatology Hospital, Zhejiang University School of Medicine, Hangzhou, 310009 China; 2 Institute of Pharmaceutics, College of Pharmaceutical Sciences, Zhejiang University, 866 Yu-Hang-Tang Road, Hangzhou, 310058 China; 3 Center for Molecular Metabolism, School of Environmental and Biological Engineering, Nanjing University of Science and Technology, 200 Xiao Ling Wei Street, Nanjing, 210094 China; 4 School of Basic Medical Sciences, Zhejiang Chinese Medical University, Hangzhou, 310053 China; 5 Department of Dermatology, Hangzhou Third People’s Hospital, Affiliated Hangzhou Dermatology Hospital, Zhejiang University School of Medicine, Hangzhou, 310009 China

**Keywords:** Cell-surface photochemistry, Ca^2+^ influx, Photonanomedicine, Anti-tumor therapy

## Abstract

Calcium (Ca^2+^) is essential for mitochondrial homeostasis and function coordination, particularly in cancer cells that metabolize frequently to sustain their growth. Photochemistry mediated calcium overload has attracted lots of attention as an effective way to achieve tumor suppression. Herein, we developed a photonanomedicine to synergistically induce calcium overload via cell-surface photochemistry and thus tumor suppression. Specifically, the photosensitizer, protoporphyrin IX (PpIX) was loaded onto upconversion nanoparticles (UCNP), which was subsequently modified by a polymer bearing photo-crosslinking cinnamate (CA) groups. The resulting nanoparticle was further functionalized by anti-CD20 aptamers (Apt), to give photonanomedicine. The interaction between CD20 receptors and anti-CD20 aptamers allowed photonanomedicine to accurately attach onto the Raji cell surface after an intravenous injection. Following the local application of a 980 nm NIR laser, the photonanomedicine was able to capture the NIR light and convert it into ultraviolet (UV) light. On one hand, the converted UV light led the crosslinking of cinnamate groups in photonanomedicine, further stimulating the clustering of CD20 receptors and causing Ca^2+^ influx. On the other hand, the UV light could simultaneously excited PpIX to generate reactive oxygen species (ROS) in situ to break down the integrity of cell membrane and lead to an influx of Ca^2+^. The synergistic Ca^2+^ overload mediated by photonanomedicine exhibited an enhanced and superior anti-tumor efficacy. We believe this photonanomedicine expands the toolbox to manipulate intracellular Ca^2+^ concentration and holds a great potential as an anti-tumor therapy.

**Supplementary Information:**

The online version contains supplementary material available at 10.1186/s12951-023-02090-z.

## Introduction

Calcium ion (Ca^2+^) regulates many fundamental physiological processes [1, 2], including, but not limited to, expression of genes, muscle contractions, metabolism, phagocytosis, apoptosis, cell division, motility. Disturbance of intracellular Ca^2+^ homeostasis, especially aberrant cytosolic accumulation of free Ca^2+^ (intracellular Ca^2+^ overload), is widely found to cause different types of cellular damage and even death [3, 4]. Notably, tumor cells, which feature a higher frequency of Ca^2+^ signaling, are more sensitive to Ca^2+^ regulation than normal cells [5, 6]. This feature might provide a new avenue for tumor treatment. Recently, the development of therapeutic agents to induce Ca^2+^ overload in tumor cells has received more and more attention [7–9].

Excessive intake of exogenous Ca^2+^ by tumor cells is a direct and effective way to induce intracellular Ca^2+^ overload [10, 11]. Certain calcium-based nanomaterials, such as inorganic CaCO_3_ [12], CaO_2_ [13], and CaP [14], are thus applied to combat tumor via intracellular Ca^2+^ overload. However, the concentration of intracellular Ca^2+^ is finely regulated by voltage-sensitive or agonist-operated calcium channels, as well as intracellular stores (mitochondria and endoplasmic reticulum (ER)). Simple addition of exogenous substances containing Ca^2+^ might not break down the intracellular Ca^2+^ homeostasis [15, 16]. Strategies targeting the meticulous regulating mechanism of intracellular Ca^2+^, which involves cell-membrane disruption, mitochondrial dysfunction, and endoplasmic reticulum buffering imbalance, are thus of great value [17–20].

Photochemistry is the study on photochemical reactions between light and molecules, which has been widely applied in biomedical field, such as phototherapy [21–23], molecular biology [24, 25], processing biomaterials [26–28], etc. Particularly, phototherapy has drawn lots of attention due to the controllability as well as effectiveness. Two prominent strategies of phototherapy are (i) a photochromic moiety is incorporated between pharmacophores, whose distance could be photomodulated and consequently lead to cluster of binding pockets, such as receptors. For example, in our previous study, multiple anti-CD20 aptamers were linked by a polymer containing photochromic moiety of cinnamic acid. After exposed to up-converted ultraviolet light, the cinnamic acids were crosslinked, and induced the clustering of CD20 receptors, which subsequently led to the influx of Ca^2+^ and cell death [29]. (ii) photodynamic therapy (PDT), which could produce reactive oxygen species (ROS) to cause damage to macromolecules, i.e. lipids, proteins and nuclear acids, and directly induce cell death [30–32]. Additionally, PDT could kill cells in an indirect way: the produced ROS interfered with the mitochondrial Ca^2+^ buffering capacity, and resulted in intracellular Ca^2+^ overload and subsequent cell death. Besides, ROS produced from cell-membrane bound photosensitizers could break down the integrity of cell membrane and lead to an influx of Ca^2+^ [33–35]. Although the aforementioned strategies have demonstrated effective in tumor therapy, the overall therapeutic efficacy needs a further improvement, especially given adequate amount of receptors on tumor cells as well as oxygen are required for these two strategies, respectively.

**Scheme 1:**
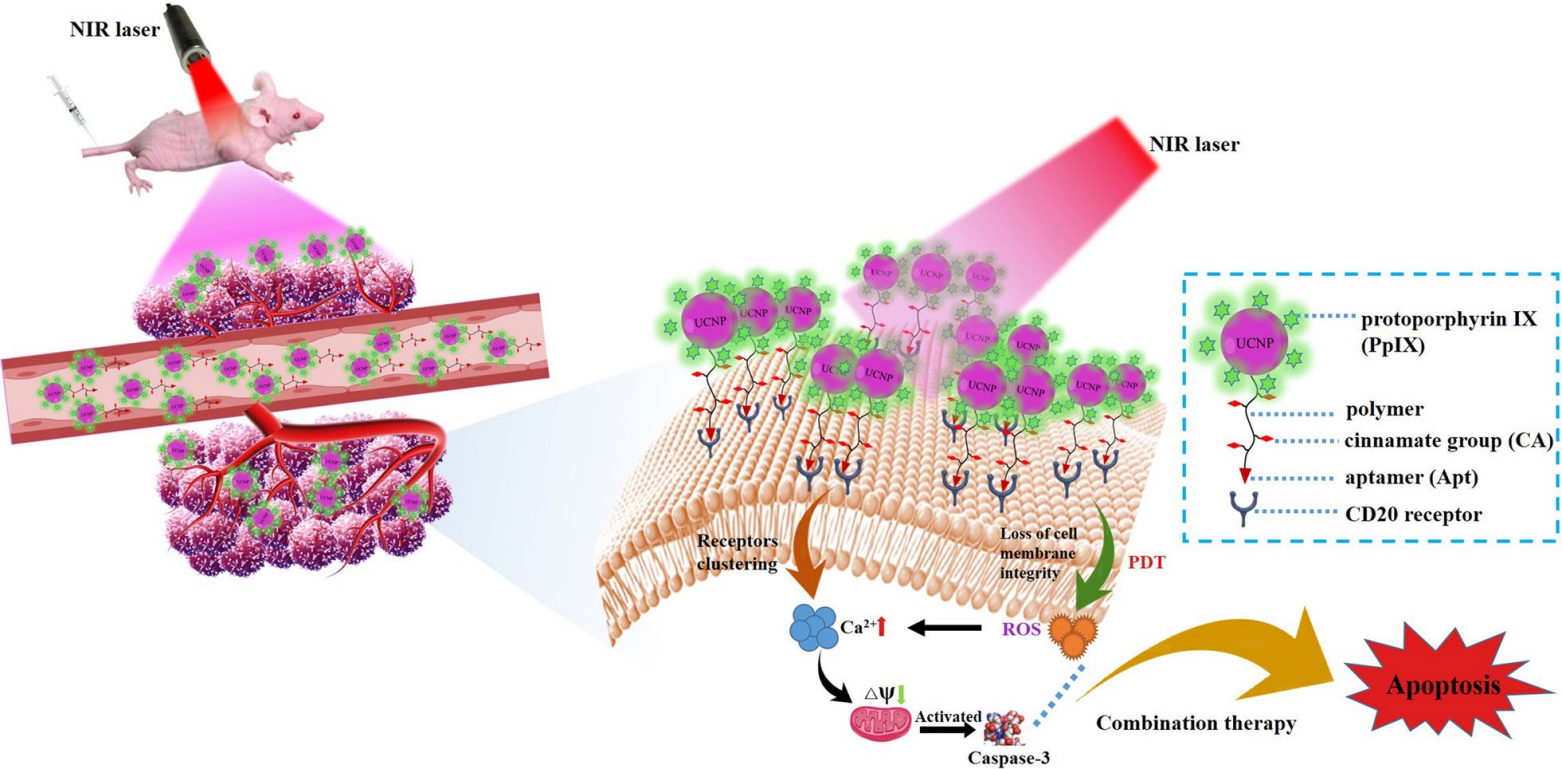
Schematic illustration of the synergistic antitumor effects of photonanomedicine via NIR upconversion controlled cell-surface receptors clustering and photodynamic therapy (PDT).

Bearing these in mind, we proposed combination of two strategies of phototherapy would contribute to an enhanced anti-tumor outcome. To this end, we developed a photonanomedicine to synergistically induce calcium overload via cell-surface photochemistry and thus tumor suppression (Scheme 1). Specifically, the photosensitizer, protoporphyrin IX (PpIX) was loaded onto upconversion nanoparticles, which was further modified by a polymer bearing photo-crosslinking cinnamate groups and anti-CD20 aptamers, to give the photonanomedicine. The interaction between CD20 receptors and anti-CD20 aptamers allowed photonanomedicine to accurately attach onto the Raji cell surface after an intravenous injection. Following the local application of a 980 nm NIR laser, the photonanomedicine was able to capture the NIR light and convert it into ultraviolet (UV) light. On one hand, the converted UV light led the crosslinking of cinnamate groups in photonanomedicine, further stimulating the clustering of CD20 receptors and causing Ca^2+^ influx. On the other hand, the UV light could simultaneously excited PpIX to generate reactive oxygen species (ROS) in situ to break down the integrity of cell membrane and lead to an influx of Ca^2+^. The synergistic Ca^2+^ overload mediated by photonanomedicine exhibited an enhanced and superior anti-tumor efficacy. We believe this photonanomedicine expands the toolbox to manipulate intracellular Ca^2+^ concentration and holds a great potential as an anti-tumor therapy.

## Results and discussions

### Synthesis and characterization of photonanomedicine

**Fig. 1:**
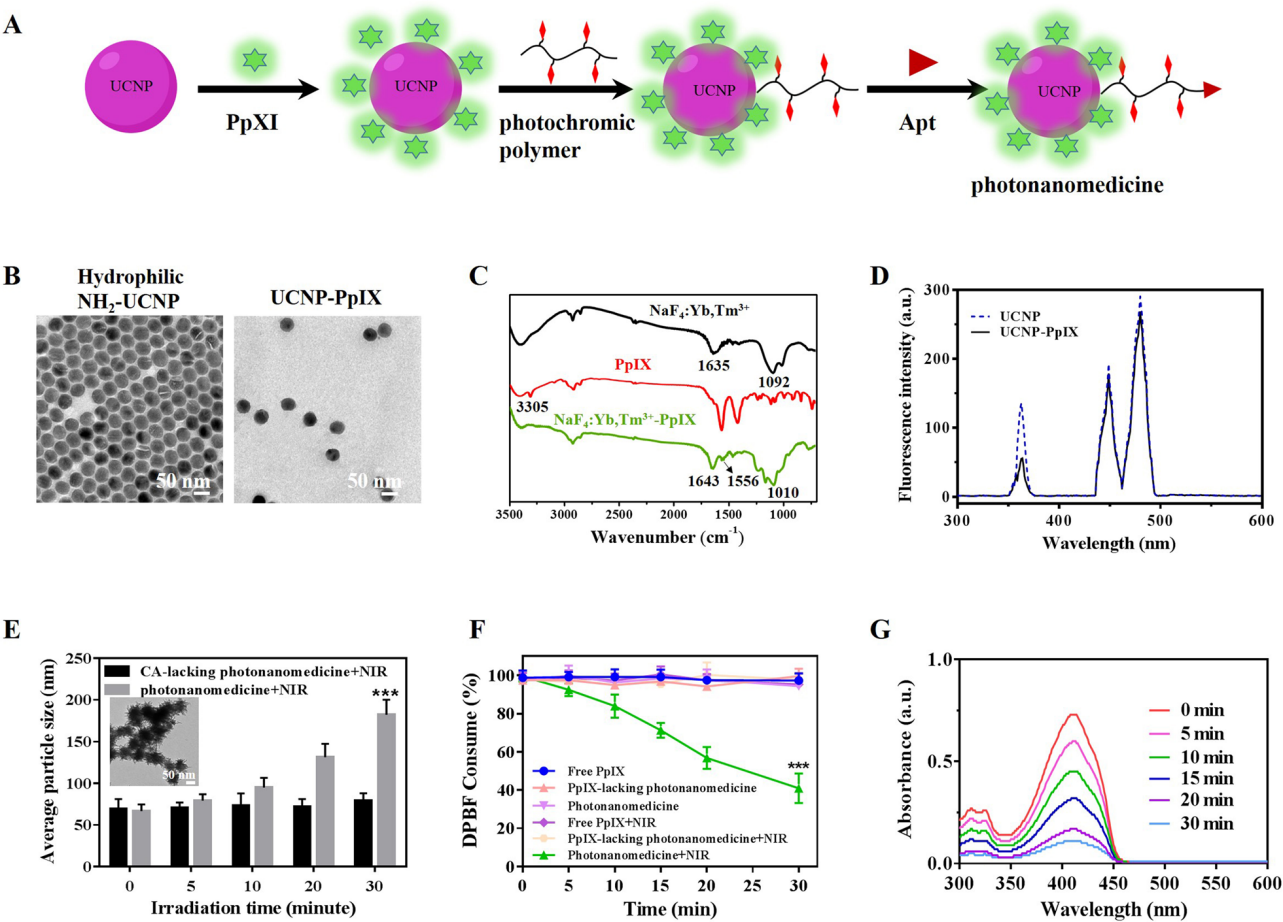
Synthesis and characterization of photonanomedicine. **A** Schematic depiction of the fabrication of photonanomedicine. **B** A representative TEM image of hydrophilic amino-functionalized UCNP and PpIX modified UCNP. **C** Fourier transform infrared (FTIR) spectra of amino-functionalized UCNP (black curve), PpIX (red curve) and UCNP-PpIX (green curve). **D** Luminescence emission spectra of UCNP (blue line) and UCNP-PpIX (black line) that were irradiated by a NIR laser. **E** Hydrodynamic sizes of CA-lacking photonanomedicine and photonanomedicine upon 980 nm NIR irradiation (2 W/cm^2^) for various time periods (insert: a representative TEM picture of photonanomedicine after 980 nm NIR irradiation for 30 min). **F** Consumption of DPBF over time after different treatments. **G** Absorbance changes of DPBF treated with photonanomedicine after 980 nm NIR laser irradiation for various time periods. Data were presented as means ± SD, ***p < 0.001, compared with five other control group

The photonanomedicine was fabricated via several procedures as indicated in Fig. 1A and Scheme S1. To begin with, the amino-modified hydrophilic UCNP was synthesized through a ligand-exchange way between oleic acid (OA)-coated NaYF_4_: Yb^3+^, Tm^3+^ (OA-UCNP [36, 37], Additional file 1: Fig. S1A–D) and 2-aminoethyl dihydrogenphosphate (AEP) according to previous reports [29, 38]. The successful exchange was verified by Fourier transform infrared (FTIR) spectroscopy (Additional file 1: Fig. S1E), and the obtained amino-modified hydrophilic UCNP displayed a uniform and spherical morphology (Fig. 1B). Then protoporphyrin IX (PpIX) was conjugated onto hydrophilic UCNP via amidation reaction between the amino groups of UCNP and the carboxyl groups of PpIX to afford UCNP-PpIX. As shown in Fig. 1C, the FTIR results showed that a new peak at 1010 cm^−1^ was appeared after conjugating of UCNP with PpIX, which was attributed to the stretching vibration of C-O-N. Besides, the amide bands C = O stretching vibration was found the absorption peak at 1643 cm^−1^, indicating the successful synthesize of UCNP-PpIX. The loading content of PpIX for UCNP was determined via the UV-vis method and calculated as 2.09%. The TEM image and dynamic light scanning (DLS) showed that the UCNP-PpIX had a uniform structure with an average diameter of 47.9 ± 3.1 nm (Fig. 1B and Additional file 1: Fig. S1F). As shown in Fig. 1D, the emission intensity of UCNP-PpIX at 365 nm decreased when compared to that of UCNP, indicating UCNP elicited luminescence could absorbed by PpIX, whose absorption wavelength was at nearly 365 nm (Additional file 1: Fig. S2). Subsequently, the photochromic polymer was synthesized via reversible addition-fragmentation chain transfer (RAFT) polymerization followed by post-polymerization modification (Additional file 1: Figs. S3 and S4) [29]. After that, the photochromic polymer was introduced onto the UCNP-PpIX through amidation reaction with remaining amino groups on UCNP. This successful modification of organic polymer onto inorganic UCNP was verified by TEM. Additional file 1: Fig. S5 evidenced a spherical core surrounded by a cloudy-like coating (organic polymer). Lastly, the thiol esters on the surface of resulting nanoparticles were reduced to afford thiol-derivatized nanoparticles, which were then functionalized with the maleimide-modified anti-CD20 aptamer (Apt) to acquire the photonanomedicine, whose structure, especially the conjugation of Apt, was confirmed by agarose gel electrophoresis as reported previously (Additional file 1: Fig. S6) [29]. The stability of photonanomedicine and corresponding intermediate nanoparticles in different medium, including phosphate buffer saline (PBS) and serum, were studied by DLS. As suggested in Additional file 1: Fig. S7, no aggregates of nanoparticles in all tested medium were detected for at least one week, demonstrating the good stability of fabricated photonanomedicines.

### Photo-crosslinking and ROS generation of photonanomedicine in vitro

We then evaluated the photo-crosslinking capacity of photonanomedicine under NIR irradiation via DLS. Figure 1E showed that with the increase of laser time, the size of photonanomedicine increased significantly after a 5-min irradiation, and reaching about 300 nm when irradiated for 30 min. This crosslinking capacity of photonanomedicine was further visualized by TEM, in which large aggregates of photonanomedicine after NIR irradiation were observed (inserted image in Fig. 1E). It is worth noting that, the NIR irradiation did not affect the particle size of CA-lacking photonanomedicine (Fig. 1E).

Then the ROS generation ability of photonanomedicine upon 980 nm NIR laser irradiation was detected by the chemical probe 1, 3-diphenylisobenzofuran (DPBF). As shown in Fig. 1F, G, the absorbance of DPBF decreased significantly with the increasing of NIR irradiation time in group of photonanomedicine, and with approximately 60% consumption in 30 min, confirming the efficient generation of ROS of photonanomedicine. While, the DPBF in other groups did not show obvious consume after NIR irradiation.

### In vitro synergistic antitumor effect mediated by photonanomedicine

The in vitro therapeutic efficiency of photonanomedicine was assessed, using PpIX-lacking or CA-lacking mono photonanomedicine as control. Firstly, the biocompatibility of these preparations was evaluated on healthy cells, including human umbilical vein endothelial cells (HUVEC), human embryonic kidney cells (HEK293) and mouse embryonic fibroblast cells (NIH3T3). No significant reduction on cell viability was observed when the concentration of tested preparations reached up to 1000 µg/mL as displayed in Additional file 1: Fig. S8. Meanwhile the biocompatibility of NIR irradiation was also confirmed, and the results showed that NIR irradiation with power density of 2 W/cm^2^ for 30 min (5 min break after 10 min of irradiation) did not affect Raji cell viability (Additional file 1: Fig. S9). The laser power was fixed at 2 W/cm^2^ in the following experiments, unless specially noted.

**Fig. 2:**
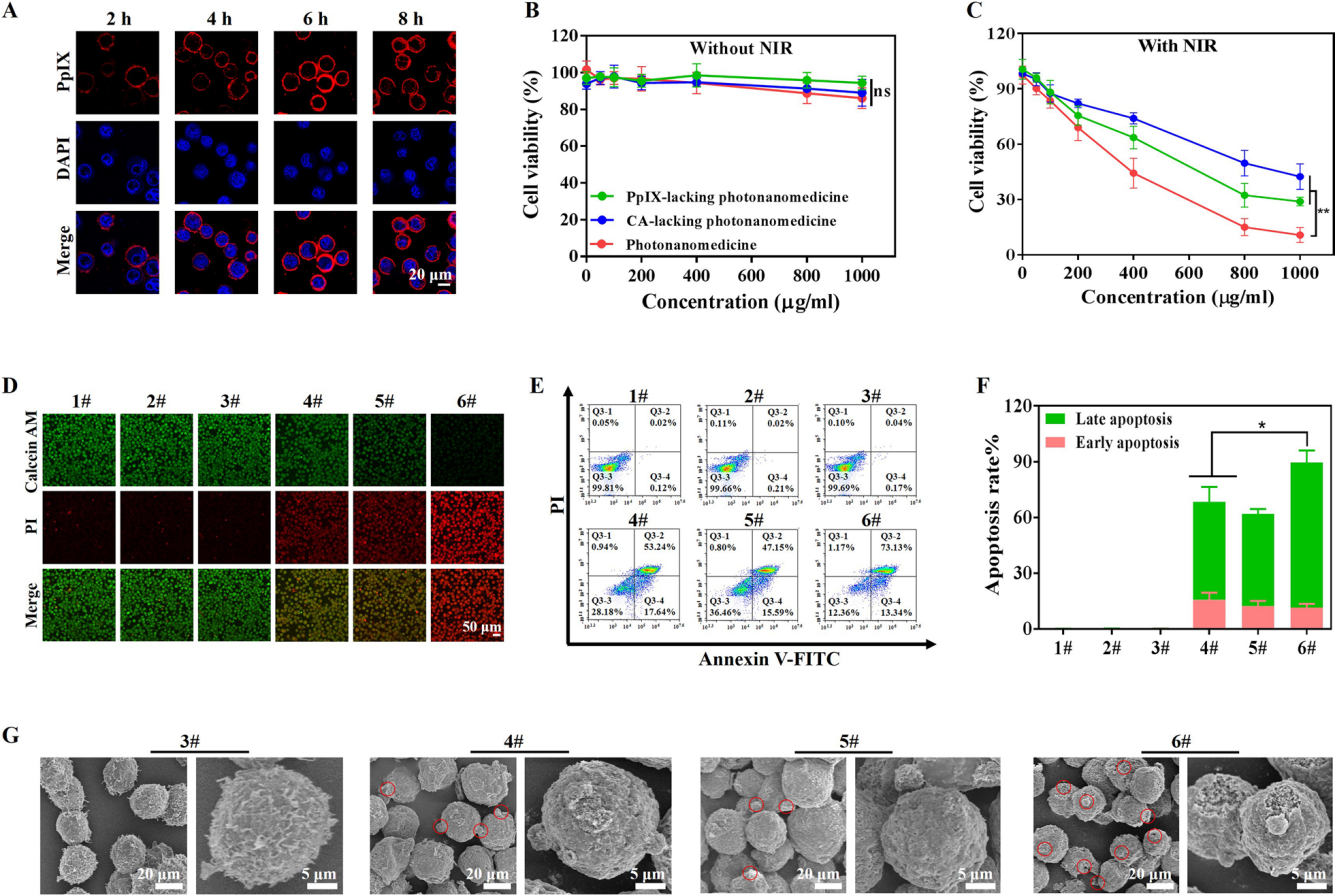
In vitro antitumor efficacy study. **A** Confocal microscopy images of Raji cells after exposure to photonanomedicine for various time periods. **B**, **C** In vitro cytotoxicity of Raji cells treated with PpIX-lacking photonanomedicine, CA-lacking photonanomedicine or photonanomedicine with or without NIR irradiation. **D** Live&dead staining fluorescence images of Raji cells after various treatments with or without NIR irradiation. **E** Cell apoptosis was evaluated after different treatments by flow cytometry. **F** Apoptosis rate of Raji cells was calculated based on part **E**. **G** SEM analysis of Raji cells apoptosis after various treatments. 1#: Control; 2#: NIR; 3#: Photonanomedicine; 4#: PpIX-lacking photnanomedicine + NIR; 5#: CA-lacking photonanomedicine + NIR; 6#: Photonanomedicine + NIR. Data were presented as means ± SD, ns means no significant difference, *p < 0.05, **p < 0.01

Then we studied the binding efficiency of photonanomedicine onto Raji cells, which were CD20-overexpressed when compared with other cancer cell lines (Additional file 1: Fig. S10). As presented in Additional file 1: Fig. S11A, B, the binding capacity of photonanomedicine onto Raji cells using CD20 receptor as the anchor was positively correlated with the concentration and time. Notably, photonanomedicine could bind onto the membrane of Raji cells and remain on the membrane without detectable internalization within ~ 8 h (Fig. 2A), which might because CD20 is a slow-internalized cell-surface receptor. In contrast, when CD20-negative tumor cells, such as Jukart, were treated with photonanomedicine, they internalized these nanomedicine, as a result of lacking CD20 anchors (Additional file 1: Fig. S12). Furthermore, the antitumor efficiency of photonanomedicine plus NIR was investigated by CCK8 assay. As shown in Fig. 2B, C, without application of NIR, all the preparations displayed negligible cytotoxicity towards Raji cells. While upon NIR irradiation, the cell viability was reduced to 27.9% and 40.3% at the concentration of 1000 µg/mL, when treated with monotherapy, including PpIX-lacking (receptor-clustering therapy) or CA-lacking photonanomedicine (PDT). In a stark contrast, photonanomedicine combining receptor-clustering therapy and PDT, displayed the best tumor-killing ability with a cell viability of 10.8%, demonstrating synergistic anti-tumor effects.

To further verify this synergistic efficiency of photonanomedicine, a calcein acetoxymethyl (AM) and propidium iodide (PI) assay was also conducted. As depicted in Fig. 2D, most cells were alive and stained by green calcein AM, when incubated with preparations without NIR irradiation. Whereas there were dead cells stained by red PI fluorescence when applied with NIR irradiation, and most dead cells were observed in photonanomedicine treated group, which was consistent with the CCK8 assay. Moreover, Raji cell apoptosis induced by different treatments was detected by flow cytometry. As displayed in Fig. 2E, F, photonanomedicine could induce highest cell apoptosis ration (~ 86.5%), compared to ~ 70.9% of PpIX-lacking photonanomedicine or ~ 62.7% of CA-lacking photonanomedicine. This superior anti-tumor capacity of photonanomedicine was further visualized by SEM. As evidenced in Fig. 2G and Additional file 1: Fig. S13, cells treated with photonanomedicine plus NIR showed the severest membrane damage, and also generated the most apoptotic bodies, compared to that of corresponding monotherapy.

### Photonanomedicine induced clustering of cell-surface receptor and PDT in vitro

**Fig. 3:**
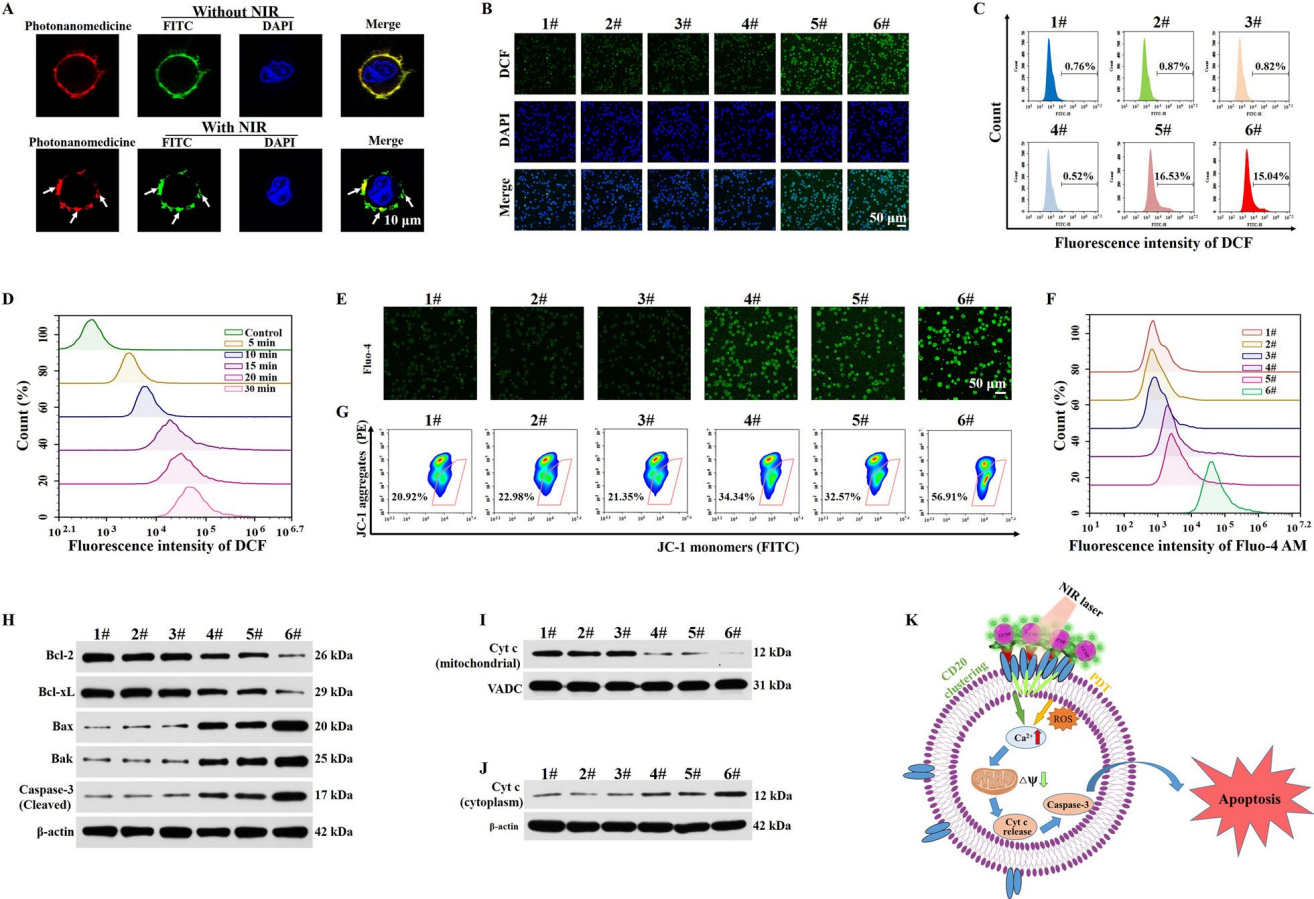
Analysis of antitumor mechanism in vitro. **A** Fluorescence images of the distribution of CD20 receptors on Raji cell surface with or without NIR irradiation. **B** Confocal microscopy images of ROS generation in Raji cells after various treatments. **C** Evaluation of intracellular ROS production using the DCFH-DA probe in Raji cells after treatment with different preparations via flow cytometry. **D** Intracellular ROS generation was detected in Raji cells treated with photonanomedicine under 980 nm NIR irradiation for various time periods. **E** Confocal microscopy images of Raji cells stained with Fluo-4 AM after different treatments. **F** The flow cytometry analysis of intracellular Ca^2+^ in Raji cells after various treatments. **G** The change of mitochondria membrane potential of Raji cells after different treatments was objectively measured using flow cytometry. **H** Expression of anti-apoptotic proteins (Bcl-2 and Bcl-xL) and pro-apoptotic proteins (Bak, Bax and Cleaved Caspase-3) of Raji cells after various treatments. **I**, **J** Immunoblot analysis of Cyt c expression in mitochondria and cytoplasm of Raji cells after various treatments. (K) Schematic diagram of the signal events involved in the apoptosis process. 1#: Control; 2#: NIR; 3#: Photonanomedicine; 4#: PpIX-lacking photnanomedicine + NIR; 5#: CA-lacking photonanomedicine + NIR; 6#: Photonanomedicine + NIR.

We investigated the anti-tumor mechanism of photonanomedicine. First, the distribution of CD20 receptors on cell surface with or without NIR irradiation was studied. As shown in Fig. 3A, after an incubation with photonanomedicine, CD20 receptors (green, stained by FITC-labeled anti-CD20 antibody) as well as photonanomedicine (red) distributed evenly on cell surface. While when the NIR was applied, CD20 microclusters were formed with the crosslinking of photonanomedicine, as suggested by the co-localization between green and red fluorescent signals.

Then we explored whether photonanomedicine could exert oxygen to produce ROS upon NIR irradiation, using DCFH-DA probe to detect the intracellular ROS level. The results suggested that the NIR irradiation alone had no impact on the production of ROS and similar behaviors were also discovered for the cells incubated with PpIX-lacking photonanomedicine under NIR irradiation. However, the fluorescence intensity was significantly increased when the cells were treated with CA-lacking photonanomedicine or photonanomedicine supplemented with NIR irradiation for 5 min, indicating that laser irradiation led to abundant ROS generation in cells (Fig. 3B, C and Additional file 1: Fig. S14). Of note, once the laser irradiation time was extended from 5 to 30 min, the fluorescence intensity was also gradually enhanced, demonstrating that ROS generation had a time-dependent manner which consistent with the behaviors in solution (Fig. 3D and Additional file 1: Fig. S15).

According to the previous reports and our group’s research [29, 39–41], the clustering of CD20 receptors at Raji cell-surface led to the aggregation of lipid rafts, followed by the activation of Src-family protein tyrosine kinases (Src-PTKs) and triggering the influx of calcium ion, which subsequently resulted in the destruction of mitochondrial membrane potential, activation of caspase-3, and thus the apoptosis. Similarly, the produced ROS via PDT could damage the cell membrane and then increase the permeability of calcium ion, inducing the mitochondria calcium overload and further activated mitochondrial-mediated apoptosis pathway (Fig. 3K). To verify the calcium overload mediated by CD20 cluster and PDT, a Fluo-4 AM probe (green fluorescence [42]) was applied to measure the intracellular level of the calcium ion after various treatments. As displayed in Fig. 3E, F and Additional file 1: Fig. S16, the groups of PpIX-lacking photonanomedicine and CA-lacking photonanomedicine upon NIR irradiation showed increased green fluorescence intensity compared with the control group, indicating the moderate influx of calcium ion. While, cells incubated with photonanomedicine supplemented with NIR irradiation exhibited highest green fluorescence intensity as a result of synergy between CD20 cluster and PDT. Further, mitochondria membrane potential was detected by flow cytometry and confocal microscopy, using the JC-1 probe [43]. As shown in Fig. 3G and Additional file 1: Fig. S17, Raji cells exposed to photonanomedicine plus NIR irradiation showed weakest red fluorescence and strongest green fluorescence intensity, indicating a largest decrease of mitochondria membrane potential, and thus the greatest damage of mitochondria.

Subsequently, we measured the downstream signals of mitochondria damage, especially the apoptosis-related proteins. As presented in Fig. 3H and Additional file 1: Fig. S18, we found photonanomedicine plus NIR treated group exhibited highest expression of pro-apoptotic proteins, such as Bax, Bak and cleaved caspase-3, and lowest expression of anti-apoptotic proteins (Bcl-2 and Bcl-xL) than the monotherapies, i.e. PpIX-lacking or CA-lacking photonanomedicine. Moreover, we measured the expression level of cytochrome c in Raji cells after various treatments. Compared with five other groups, the expression of cytochrome c in the cytoplasm was significantly increased after photonanomedicine plus NIR treatment, along with a corresponding decline of cytochrome c in the mitochondria-revealing the release of cytochrome c from mitochondria into the cytoplasm as a result of mitochondria damage (Fig. 3I, J and Additional file 1: Fig. S19). Taken together, all the above results demonstrated that the combined therapy could remarkably increase calcium ion influx than that of monotherapy, followed by severe damage of mitochondria membrane and activation of pro-apoptotic proteins.

### Biodistribution of photonanomedicine in tumor-bearing mice after an intravenous injection

**Fig. 4:**
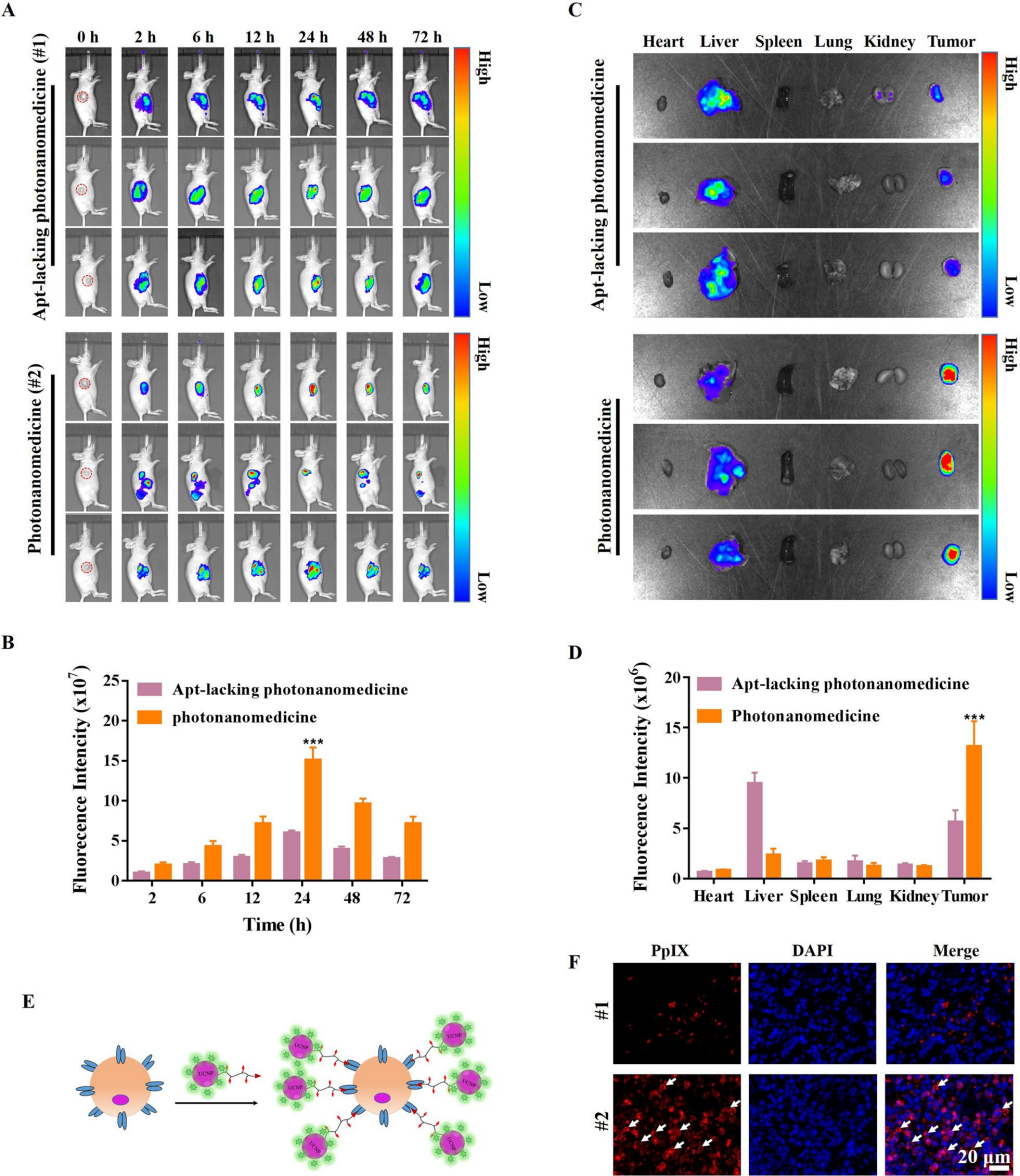
Evaluation on targeting function of photonanomedicine. A Fluorescence images of tumor-bearing mice after intravenous injection of Apt-lacking photonanomedicine and photonanomedicine. The red circle marked the tumor site. **B** The semi-quantitative analysis of fluorescence intensity in tumor sites based on part A. Fluorescence photographs **C** and mean fluorescence intensity **D** of tumor tissues and other major organs at 72 h after intravenous injection. **E** Schematic illustration of photonanomedicine targeting the Raji cell surface. **F** The distribution of Apt-lacking photonanomedicine and photonanomedicine in tumor tissues at 72 h post-i.v. injection. Data were presented as means ± SD, ***p < 0.001, compared with the corresponding control group

Next, the biodistribution of photonanomedicine after systemic administration was investigated in Raji tumor-bearing mice. As displayed in Fig. 4A, B, photonanomedicine accumulated at tumor sites in a time-dependent manner, reaching the maximum amount at 24 h post- i.v. injection. Notably, the accumulated photonanomedicine could retain in tumor for at least 72 h. In contrast, Apt-lacking photonanomedicine displayed the weaker tumor-accumulating capacity. Further the major organs, including heart, liver, spleen, lung and kidney, were collected at 72 h post-i.v. injection, and examined by In Vivo Imaging system. As displayed in Fig. 4C, D, the fluorescent intensity of tumors from photonanomedicine treated group was much higher than that of Apt-lacking photonanomedicine, further indicating the improved accumulation of photonanomedicine in the tumor site mediated by the Apt active targeting. Moreover, to explore whether the accumulated photonanomedicine could target the cell-surface CD20 of Raji cells in vivo, the tumor slices were prepared and observed via fluorescent microscopy. As expected, the results showed that photonanomedicine could precisely bound onto tumor cell membrane (white arrows), while most of Apt-lacking photonanomedicine was internalized by tumor cells (Fig. 4E, F).

### In vivo antitumor efficacy of photonanomedicine

**Fig. 5:**
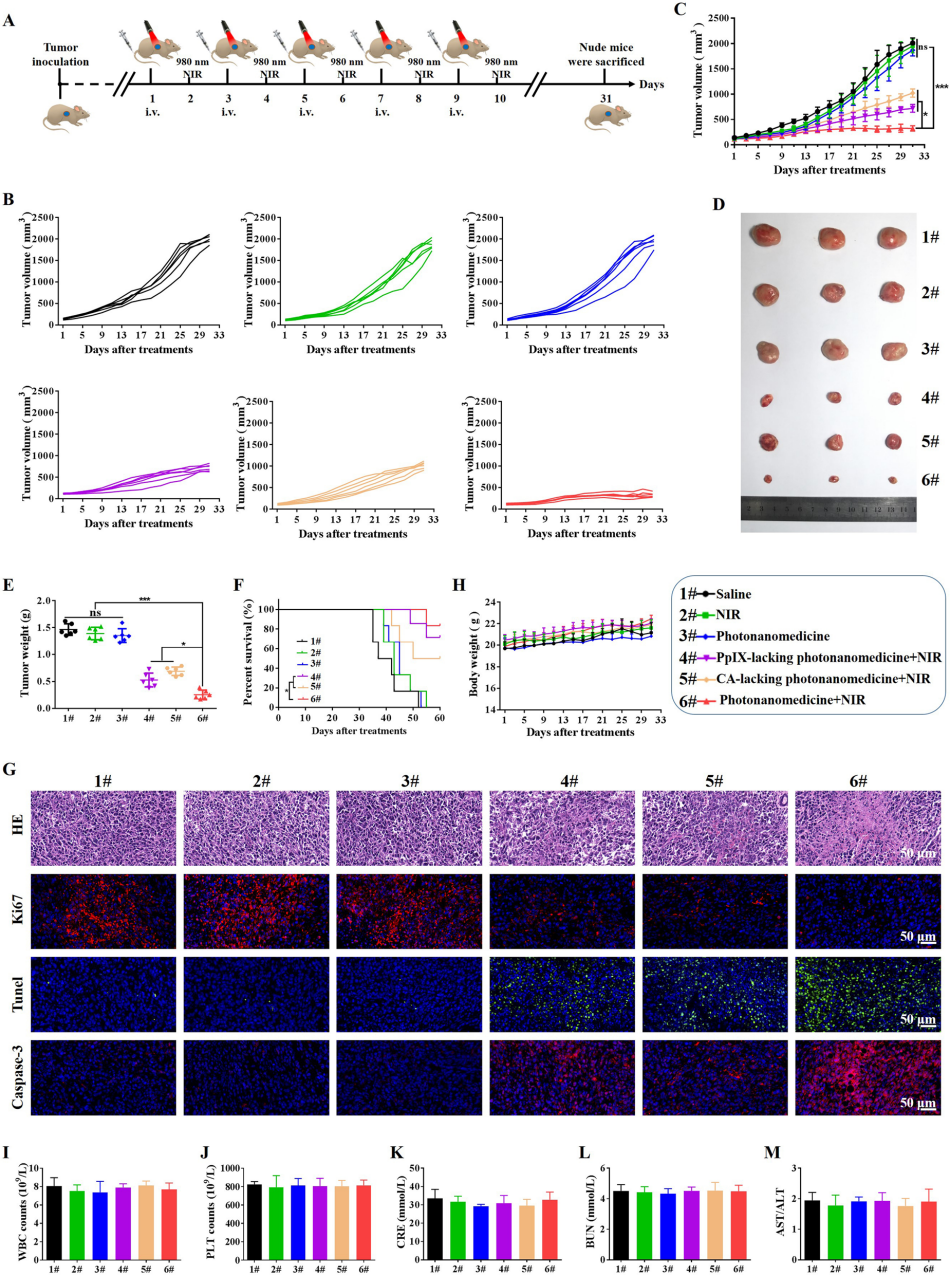
In vivo antitumor efficacy of photonanomedicine via intravenous injection. **A** Schematic depiction of detailed treatments. **B**, **C** Tumor growth curves after different treatments. **D** Digital photographs of excised tumor tissues from representative mice after various treatments. **E** Tumor weights of mice at the end time points. **F** Survival curves of mice after various treatments. **G** Representative photographs of tumor sections stained by H&E, Ki67, Tunel and Casepase-3 after various treatments. **H** Weight change curves of mice after different treatments. **I**-**M** Blood biochemical indexes of white blood cells (WBC), platelets (PLT), creatinine (CRE), blood urea nitrogen (BUN) and the ration of aspartate transaminase (AST)/alanine aminotransferase (ALT). Data were presented as means ± SD, ns means no significant difference, *p < 0.05, ***p < 0.001

The combined therapeutic efficiency of photonanomedicine was explored in Raji tumor-bearing mice, and the treatment regimen was presented in Fig. 5A. Tumor-bearing mice were randomly divided into 6 groups (n = 6), and intravenously receiving different agents as following: (1) Saline; (2) NIR laser; (3) Photonanomedicine; (4) PpIX-lacking photonanomedicine + NIR laser (cell-surface receptors clustering monotherapy); (5) CA-lacking photonanomedicine + NIR laser (PDT monotherapy); (6) Photonanomedicine + NIR laser (combination therapy). Notably, in situ NIR irradiation was performed at 24 h post-i.v. injection.

The tumor growth curve for each mouse after receiving corresponding treatment was displayed in Fig. 5B. All the tumors in mice receiving photonanomedicine plus NIR grew slowest with a tumor inhibition rate of ~ 83.8% (Fig. 5C). In a stark contrast, the monotherapies, i.e. PpIX-lacking photonanomedicine + NIR laser and CA-lacking photonanomedicine + NIR laser, displayed a moderate tumor inhibition rate of 66.4% and 50.2%, respectively (Fig. 5C). Moreover, images along with weights of tumors collected from mice treated with various formulations further verified the synergistic anti-tumor capacity of photonanomedicine plus NIR (Fig. 5D, E). Besides, the survival time of mice treated with photonanomedicine plus NIR was also significantly prolonged compared to that of corresponding monotherapies, further confirming the synergy (Fig. 5F). Additionally, we conducted the H&E analysis for collected tumor tissues. Most karyorrhexis (fragmentation) and karyolysis (dissolution) were observed in the tumor from mice administrated with combined therapeutic photonanomedicine plus NIR, followed by the monotherapies, including PpIX-lacking photonanomedicine + NIR laser and CA-lacking photonanomedicine + NIR laser. Similarly, Ki67, Tunel and Caspase-3 analysis suggested photonanomedicine plus NIR induced the largest proportion of cell apoptosis and the least cell proliferation (Fig. 5G).

Next, we studied the biocompatibility of photonanomedicine in vivo. No significant loss of body weights for mice in all groups were observed, suggesting the safety of applied formulations (Fig. 5H). Moreover, the H&E staining of major organs, including heart, liver, spleen, lung and kidney, showed that no obvious histopathological abnormalities, degeneration, or lesions, occurred during various treatments (Additional file 1: Fig. S20). In addition, we found the level of cellular components in blood, including white blood cells (WBC) and platelets (PLT), remained stable after receiving treatments (Fig. 5I, J). Meanwhile, the functions of kidney (indicated by serum creatinine (CRE), and blood urea nitrogen (BUN)) and liver (indicated by the ration of aspartate transaminase (AST)/alanine aminotransferase (ALT)) were not affected after administrated with various treatments (Fig. 5K, L and M).

## Conclusions

In summary, a synergistic photonanomedicine based on cell-surface receptors clustering and PDT was developed for secure and efficient tumor therapy. The constructed photonanomedicine could precisely targeted the cell-surface via the tight binding between anti-CD20 aptamers and overexpressed CD20 receptors on the tumor cell membrane. After a local application of NIR laser, the UCNP in photonanomedicine acted as NIR sensors to shift NIR light to UV light, which then lead to the crosslinking of photochromic polymer in photonanomedicine as well as exciting photosensitizer in photonanomedicine to generate abundant ROS at cell surface. The clustered CD20 receptors led by crosslinking of photochromic polymer along with damaged cell membrane caused by ROS, resulted in the overload of intracellular Ca^2+^ and consequently cell apoptosis. This cell-surface photochemistry mediated synergistic therapeutic system exhibited remarkable antitumor efficiency both in vitro and in vivo, and without causing any apparent toxicity. Therefore, our study provides a cell-surface photochemistry mediated anti-tumor nanomedicine with high selectivity and enhanced therapeutic efficacy.

### Supplementary Information

**Additional file 1.** Additional materials and methods section, scheme and figures.

## Data Availability

All data and materials during this study are present in the paper and/or the Supporting Information. Additional data related to this study are available from the corresponding authors upon reasonable request.
