# Supplementary material for: Cell-surface photochemistry mediated calcium overload for synergistic tumor therapy
Source: J Nanobiotechnology. 2023 Sep 19;21:335. doi: 10.1186/s12951-023-02090-z (PMC10510147; doi:10.1186/s12951-023-02090-z)
Supplement: Supplementary file 1 — Additional file 1. Additional materials and methods section, scheme and figures. [file 12951_2023_2090_MOESM1_ESM.docx]

**Additional file 1**

**Cell-surface photochemistry mediated calcium overload for synergistic tumor therapy**

Jun Wang, ^1,#^ Wei Wang, ^1,#^ Qingmei Shen^4^, Lan Lan^5,^ Cuiping Guan^5^, Xinchang Xu, ^1,*^ Weishuo Li, ^3,*^ and Yongzhong Du ^2,*^

^1^ Department of Pharmacy, Hangzhou Third People’s Hospital, Affiliated Hangzhou Dermatology Hospital, Zhejiang University School of Medicine, Hangzhou, 310009, China

^2^ Institute of Pharmaceutics, College of Pharmaceutical Sciences, Zhejiang University, 866 Yu-Hang-Tang Road, Hangzhou, 310058, China

^3^ Center for Molecular Metabolism, School of Environmental and Biological Engineering, Nanjing University of Science and Technology, 200 Xiao Ling Wei Street, Nanjing, 210094, China

^4^ School of Basic Medical Sciences, Zhejiang Chinese Medical University, Hangzhou, 310053, China

^5^ Department of Dermatology, Hangzhou Third People’s Hospital, Affiliated Hangzhou Dermatology Hospital, Zhejiang University School of Medicine, Hangzhou, 310009, China

^#^ These authors contributed equally to this work.

Corresponding Authors:

Yongzhong Du, E-mail: [duyongzhong@zju.edu.cn](mailto:duyongzhong@zju.edu.cn), Phone: +86-571-88208435

Xinchang Xu, E-mail: zxyyadr@163.com

Weishuo Li, E-mail: liweishuo@njust.edu.cn

**Contents**

**Experiment**

**Scheme S1.** Illustration of the synthesize procedure of photonanomedicine.

**Fig. S1.** Characteristic of UCNP and PpIX-modified UCNP.

**Fig. S2.** The absorption spectrum of the PpIX.

**Fig. S3.** Synthetic and ^1^H-NMR spectrum of the polymer.

**Fig. S4.** Synthetic and ^1^H-NMR spectrum of photochromic polymer.

**Fig. S5.** A representative TEM photograph and size distribution of Apt-lacking photonanomedicine.

**Fig. S6.** A representative image of agarose gel electrophoresis for FITC tagged Apt-lacking photonanomedicine (i), mixture of Apt-lacking photonanomedicine and aptamer (ii), and photonanomedicine (iii).

**Fig. S7.** The stability of photonanomedicine and corresponding intermediate nanoparticles in different medium.

**Fig. S8.** Cell viability of HUVEC, HEK293 and NIH3T3 cells after incubation with photonanomedicine at various concentrations for 48 h.

**Fig. S9.** Cell viability of Raji cells after irradiation with 980 nm NIR laser (2 W/cm^2^) under different irradiation time.

**Fig. S10.** CD20 expression levels of various cancer cell lines.

**Fig. S11.** Flow cytometry analysis of Raji and Jurkat cells incubated with photonanomedicne at different concentrations and time points, respectively.

**Fig. S12.** Confocal microscopy images of Jurkat cells after exposure to photonanomedicine for various time periods.

**Fig. S13.** SEM images of Raji cells without crosslinking of photonanomedicine at cell surface.

**Fig. S14.** The mean fluorescence intensity of DCF was quantitatively calculated by Image J based on Fig. 3B.

**Fig. S15.** Intracellular ROS generation in Raji cells was measured via confocal microscopy after treatment with photonanomedicine for various time periods.

**Fig. S16.** The mean fluorescence intensity of Fluo-4 AM was quantitatively calculated by Image J based on Fig. 3E.

**Fig. S17.** Fluorescence images of Raji cells stained with JC-1 after various treatments (A) and the red/green fluorescence intensity ration of JC-1was analyzed via Image J (B).

**Fig. S18.** Quantitatively analysis of relative protein expression of Bcl-2, Bcl-xL, Bax, Bak and Caspase-3 in Raji cells after various treatments based on Fig. 3H.

**Fig. S19.** Quantitatively analysis of relative Cyt c protein expression in Raji cells after various treatments based on Fig. 3I and J.

**Fig. S20.** Confocal Microscopy images of H&E stained major organs (heart, liver, spleen, lung and kidney) of mice with various treatments via intravenous injection at the end of observation.

**Materials and methods**

**Materials and animals**

Rare earth chlorides (YCl_3_·6H_2_O, YbCl_3_·6H_2_O, TmCl_3_·6H_2_O), NH_4_F and NaOH were obtained from Macklin Biochemical Co., Ltd (Shanghai, China). 1-octadecene, oleic acid and 2-aminoethyl dihydrogenphosphate (AEP) were purchased from Sigma-Aldrich Co., Ltd (St. Louis, MO, USA). 1-ethyl-3-(3-dimethylaminopropyl)carbodiimide (EDC), N-hydroxysuccinimide (NHS) and protoporphyrin IX (PpIX) were provided by Aladdin Co., Ltd (Shanghai, China). N,N’-dicyclohexylcarbodimide (DCC), 4-dimethylaminopyridine (DMAP), 2-hydroxyethyl methacrylate (HM), 2-cyano-2-propyl benzodithioate and 4.4’-azobis(4-cyanovaleric acid) (ACVA) were acquired from Sigma-Aldrich Co., Ltd (St. Louis, MO, USA). Ethanol absolute, cyclohexane, methanol, cinnamic acid and dimethyl sulfoxide (DMSO) were obtained from Aladdin Co., Ltd (Shanghai, China).The Mal-PEG_3_-Mal was synthesized by ToYang Bio Tech. Inc (Shanghai, China). Dulbecco's Modified Eagle Medium (DMEM) and Roswell Park Memorial Institute-1640 (RPMI-1640) was provided by Sigma-Aldrich Co., Ltd (St. Louis, MO, USA). Cell counting kit-8 (CCK8), 3-(4,5-dimethyl-2-thiazolyl)-2,5-diphenyl-2H-tetrazolium bromide (MTT), Annexin V-FITC/PI apoptosis detection kit, Fluo-4 AM and mitochondrial membrane potential assay kit (JC-1) were purchased from Beyotime Biotech Co., Ltd (Shanghai, China). The anti-CD20 aptamer (sequence: 5’-CTCCTCTGAC TGTAACCACG CCGTATGTCC GAAATACGGA GAACAGCACT CATATGCAAG CCATACGCGG AGGTGCACGC GCATAGGTAG TCCAGAAGCC-3’) was provided by Sangon Biotech Co., Ltd (Shanghai, China). Primary antibodies including anti-CD20, anti-Bcl-2, anti-Bcl-xL, anti-Bak, anti-Bax, anti-cleaved caspase-3 were all obtained from Abcam Co., Ltd (Cambridge, UK).

Balb/c nude mice aged 6-8 weeks (18-20 g) were purchased from Slack Laboratory Animal Co., Ltd (Shanghai, China). All animal experiments were performed in accordance with the National Institutes of Health Guide for the Care and Use of Laboratory Animals with the approval of the Scientific Investigation Board of Zhejiang University.

**Preparation and characterization of UCNP-PpIX**

In a proper route of NaYF_4_:Yb^3+^,Tm^3+^ upconversion nanoparticles (UCNP) preparation, 0.795 mmol YCl_3_**·**6H_2_O, 0.2 mmol YbCl_3_**·**6H_2_O and 0.005 mmol TmCl_3_**·**6H_2_O were weighted into a 50 mL flask. Subsequently, 6 mL of oleic acid (OA) and 15 mL of octadecene were added, and the reaction system was heated to 160 °C for 40 min to form a homogeneous solution. When the solution cooled down to room temperature, the mixture of NaOH (2.5 mmol) and NH_4_F (4 mmol) in 10 mL of methanol was added and the reaction system was kept at 50 °C for 30 min with magnetic stirring. After that, methanol was evaporated and the mixture solution was heated to 300 °C for 1.5 h under argon protection. Finally, the mixture solution was washed with ethanol for at least three times, and UCNP was collected via centrifugation (4500 rpm, 5min) and stored in cyclohexane.

Then the amino-functionalized UCNP was further fabricated via ligand exchange. Briefly, 200 mg of 2-aminoethyl dihydrogenphosphate (AEP) was dispersed in 10 mL of a mixture of deionized water and ethanol with a volume ration of 3:2, and 5 mL of cyclohexane solution of OA-functioned UCNP was dropped into the system and stirred vigorously for 48 h at room temperature. Afterward, the UCNP could transfer from top cyclohexane to bottom water layer and be collected via centrifugation (4500 rpm, 5min) for further use.

In order to covalently conjugated protoporphyrin IX (PpIX) to UCNP, 5 mL of DMSO solution containing 5 mg (0.0089 mmol) of PpIX, 8.28 mg (0.043 mmol) of 1-ethyl-3-(3-dimethylaminopropyl)carbodiimide (EDC), and 6.14 mg (0.053 mmol) of N-hydroxysuccinimide (NHS) was stirred at 60 °C for 1 h, and then 5 mL of amino-functionalized UCNP (2 mg/mL) was added into the solution and stirred vigorously for another 48 h. Afterwards, UCNP-PpIX conjugates were centrifuged and washed with water to remove any unreacted PpIX. The amount of photosensitizer attached to UCNP was calculated from the PpIX absorption spectrum. The Fourier transform infrared spectroscopy (FTIR) absorption of amino-functionalized UCNP, PpIX and UCNP-PpIX was inspected by Nicolet iS50 ThermoFisher infrared spectrometer. Upconversion emission spectra of UCNP and UCNP-PpIX were performed through the fluorescence spectrometer with 980 nm laser irradiation.

**Synthesizes and characterization of** **photochromic polymer**

Our previously developed method was used to synthesize photochromic polymer. In a typical procedure, 0.423 g of 2-cyano-2-propyl benzodithioate, 0.01074 g of 4,4'-azobis(4-cyanovaleric acid), 0.95 mL of 2-hydroxyethyl methacrylate and 2.56 mL of dioxane were added into a 25 mL three-necked flask. Subsequently, argon was pumped for 1 h to remove the oxygen from the system. After that, the flask was immersed into an oil bath which had been preheated to 80 °C. The reaction system was then polymerized for 8 h under argon protection and rapidly cooled to room temperature in liquid nitrogen. Finally, the mixture solution was precipitated in 10-fold excess volume ether. The obtained precipitate was then washed thrice with ether and dried in a vacuum oven at 50 °C for 24 h, namely poly(2-hydroxyethyl methacrylate) (polymer). Next, the cinnamic acid (CA) was introduced onto polymer via esterification reaction. Briefly, 17.63 mg of CA, 122.89 mg of DCC and 7.28 mg of DMAP (CA: DCC: DMAP=1: 5: 0.5, mol: mol) were dissolved in 5 mL of anhydrous DMSO and stirrer at 50 °C for 1.0 h. Afterwards, 2 mL of anhydrous DMSO containing 50.82 mg of polymer was added to the solution and stirred at 60 °C for another 48 h. The obtained solution was then dialysis against deionized water with a dialysis membrane (MWCO: 3.5 kDa) for 48 h, followed by lyophilization and the photochromic polymer was obtained. The ^1^H-NMR spectra of the polymer and photochromic polymer were acquired via an NMR spectrometer (AC-80, BrukerBioSpin, Germany).

**Preparation and characterization of Apt-lacking photonanomedicine**

The remaining amino groups of UCNP-PpIX was used to react with the carboxyl groups of photochromic polymer to obtain the Apt-lacking photonanomedicine. In a typical procedure, 50 mg of photochromic polymer was weighted into a 25 mL three-necked flask and dissolved in 5 mL of anhydrous DMSO. Subsequently, 24.3 mg of EDC and 17.9 mg of NHS was added and the reaction system was stirrer at 50 °C for 1.0 h. After that, 10 mg of UCNP-PpIX dispersed in 5 mL of DMSO was dropped into the system and the mixed solution was stirred at 60 °C for another 48 h. The product was then centrifuged (14000 rpm, 15 min) to acquire the precipitate. Finally, the precipitate was washed with deionized water for three times and dried at 50 °C to obtain the Apt-lacking photonanomedicine. The size distribution and morphology of Apt-lacking photonanomedicine was observed via dynamic light scattering (DLS) and transmission electron microscope (TEM), respectively.

**Synthesize and characterization of photonanomedicine**

Briefly, 20 mg of Apt-lacking photonanomedicine was charged in a 10 mL three-neck flask and dispersed in 3 mL of distilled THF. Thereafter, 200 μL of cyclohexylamine was added and the reaction system was stirred for 48 h with the protection of argon at room temperature. After that, the mixture solution was centrifuged (14000 rpm, 15 min) to acquire the precipitate. Then the precipitate was washed thrice with n-hexane and dried to obtain the thiol-derivatized Apt-lacking photonanomedicine. Next, 2 mL of Tris-EDTA (TE) buffer contained 0.02 μmol of thiol-modified aptamer was dropped into 1 mL of TE buffer which contained 0.1 μmol of Mal-PEG_3_-Mal. And the reaction system was stirred at 37 °C for 12 h, followed by dialysis against deionized water for 48 h to remove any unreacted regents (MWCO: 3.5 kDa). Finally, the acquired maleamide-functionalized aptamer solution was mixed with thiol-derivatized Apt-lacking photonanomedicine (2 mg/mL) and stirred for another 24 at room temperature to obtain photonanomedicine.

To prove the successful modification of aptamer to thiol-derivatized Apt-lacking photonanomedicine, agrose gel electrophoresis was carried out. In a typical experiment, Apt-lacking photonanomedicine was first labeled by FITC. Briefly, 200 μg of FITC was dissolved in 1 mL of ethanol and then added dropwise into the 5 mL of deionized water which contained Apt-lacking photonanomedicine (1mg/mL). After that, the mixture solution was stirred for 24 h at room temperature and the obtained solution was then centrifuged (14000 rpm, 15 min) to acquire the precipitate, which was FITC-labeled Apt-lacking photonanomedicine. Thereafter, the obtained FITC-tagged Apt-lacking photonanomedicine was reacted with malemide-functionalized aptamer to give FITC-labeled photonanomedicine. Finally, Apt-lacking photonanomedicine, photonanomedicine, and the mixture of aptamer and Apt-lacking photonanomedicine was subjected to 1% agrose gel. The obtained gel was visualized via Gel Imager (Gel Logic 200, Kodak, USA).

**Stability evaluation of nanoparticles**

To detect the stability of nanoparticles, photonanomedicines and corresponding intermediate nanoparticles were dispersed in PBS, PBS supplemented with 10% fetal bovine serum (FBS) and acid PBS (pH6.8) (1 mg/mL), respectively. Afterwards, the particle size of nanoparticles were measured by DLS instrument at predetermined time points.

**Photo-crosslinking of photonanomedicine in vitro**

Briefly, the nanoparticles of photonanomedicine or CA-lacking photonanomedicine dispersed in deionized water (800 μg/mL) were irradiated by a 980 nm NIR laser (2 W/cm^2^) for different times (0, 5, 10, 20 and 30 min). Afterwards, the particle sizes of the nanoparticles were measured via DLS, and the morphology of photonanomedicine irradiated for 30 min was observed via TEM.

**Detection of ROS generation in vitro**

The generation of extracellular ROS was measured with DPBF probes. In a typical process, DPBF was dissolved in ethanol and diluted to 10 mM, which was then mixed with 2 mL of PpIX-lacking photonanomedicine or photonanomedicine nanoparticles aqueous dispersion (0.5 mg/mL). Afterwards, the mixture was irradiated by a 980 nm NIR laser (2 W/cm^2^) for various time periods in the dark. The generation of ROS was demonstrated via the characteristic absorption decrease of the DPBF at 410 nm using a UV-vis absorption spectrum.

**In vitro synergistic antitumor effect mediated by photonanomedicine**

**Cell lines and culture**

Raji and Jurkat cells in RPMI-1640 medium supplemented with 10% (v/v) FBS and penicillin/streptomycin (100 U/mL of each) were incubated in the cell incubator (37 °C and 5% CO_2_). For HUVEC, HEK293 and NIH3T3 cell lines, the condition of culture was almost the same, expect DMEM instead of RPMI-1640 medium was used.

**Biocompatibility of photonanomedicine**

The biocompatibility of photonanomedicine without NIR irradiation was investigated by MTT assay. Briefly, HUVEC, HEK293 and NIH3T3 cells were suspended in serum-free medium and seeded into a 96-well plate with a density of 1×10^4^ cells per well for 24 h, and then the cell culture medium was refreshed with 200 μL of medium containing photonanomedicine at various concentrations ranging from 0 to 1000 μg/mL. The cells were incubated for another 48 h. After that, 20 μL of MTT were added and incubated for another 4 h at 37 °C. Subsequently, the medium was replaced with 100 μL of DMSO. Finally, the absorbance of the solution in each well was measured by microplate reader at the wavelength of 570 nm.

**Cytotoxicity of 980 nm NIR laser with different laser power**

In a typical process, Raji cells were seeded in a 96-well plate at a density of 5×10^3^ cells per well. Then the cells were exposed to 980 nm NIR irradiation at different power for 30 min (5 min break after 10 min of irradiation). Finally, cell viability was measured after 48 h incubation via CCK-8 assay, following the manufacture’s protocol.

**Expression levels of CD20 protein in tumor cell lines**

Western blot was employed to measure the CD20 expression levels in Raji, Jurkat, CT26, A549, Bel-7402 and 4T1 cell lines. In a typical experiment, Raji cells were suspended in serum-free medium and seeded in 6-well plate at a density of 5×10^5^ cells per well and allowed to cultivate overnight. Afterwards, cells were harvested and lysed by cell lysis buffer. The obtained proteins were then subjected to SDS-PAGE and immunoblotted with anti-CD20 antibody, according to the manufactory’s protocol. The β-actin was used as the reference. Afterwards, the blots were washed and incubated with horseradish peroxidase-labeled goat anti-rabbit IgG for 2 h at room temperature, followed by imaging (Chemiluminescence imager, Bio-Rad, USA).

**Cellular uptake**

Firstly, the flow cytometry was used to examine the binding efficiency of photonanomedicine onto Raji cells. In a typical experiment, Raji cells were seeded in a 1.5 mL sterile tubes at a density of 1×10^5^ cells per tube for 24 h, and then exposed to various concentration of photonanomedicine (0, 50, 100, 200, 400, 600, 800 μg/mL). After 2 h co-incubation at 37 °C, the cells were harvested and washed thrice with cold PBS. Then the fluorescence intensity was measured via flow cytometry (Cytomic FC 500MCL, Beckman, USA). Additionally, the impact of incubation time on binding was also measured. Briefly, Raji cells were seeded in a 1.5 mL sterile tubes at a density of 1×10^5^ cells per tube for 24 h, and then exposed to photonanomedicine (600 μg/mL) for different time points(0, 1, 2, 4, 6 and 8 h). Finally, the cells were harvested and the fluorescence intensity was measured via flow cytometry. Jurkat cells were served as the negative control.

Furthermore, the confocal microscopy was employed to observe the distribution of photonanomedicine. Briefly, Raji cells were seeded in 1.5 mL sterile tubes with a volume of 100 μL, and the density of the cells was set at 1×10^6^/mL. Afterwards, photonanomedicine was added into the tubes at the final concentration of 600 μg/mL and co-incubated for various time periods (2, 4, 6 and 8 h). The cells were then collected, washed thrice with cold PBS and fixed. Finally, the obtained cells were observed using confocal laser scanning microscopy (Leica, TCS SP8, Germany). Jurkat cells were served as the negative control.

**Cytotoxicity and apoptosis**

The antitumor effects of photonanomedicint performed with or without NIR irradiation were determined by CCK8 assay and live/dead staining. For the analysis of cell viability via CCK8 method, Raji cells were seeded in a 96-well plate at a density of 5×10^3^ cells per well and allowed to cultivate overnight. Afterwards, the cells were treated with PpIX-lacking photonanomedicne, CA-lacking photonanomedicne or photonanomedicine at various concentration (at a final concentration from 0 to 1000 μg/mL) for 6 h. The NIR treated groups was then irradiated with a 980 nm NIR laser (2 W/cm^2^, 5 break after 10 min of irradiation) for 30 min, followed by incubation at 37 °C for another 24 h. Cell viability was measured via CCK8 test kit, following the manufacture’s instruction. For live/dead staining, Raji cells were inoculated into a 24-well plate at a density of 1×10^4^ cells per well, incubated with the above mentioned three kinds of preparations for 6 h. Afterwards, the cells were irradiated with a 980 nm NIR laser at 2 W/cm^2^ for 30 min. Next, the cells were incubated with calcein AM (4 μM) and PI (4 μM) for 30 min. Finally, the labeled cells were observed using a confocal microscopy.

Cell apoptosis induced by photonanomedicne with or without NIR irradiation were evaluated by flow cytometry. In a typical procedure, Raji cells were seeded in a 12-well plate with the cell density of 1×10^5^ cells per well and allowed to cultivate overnight. After that, cells were exposed to PpIX-lacking photonanomedicne, CA-lacking photonanomedicne or photonanomedicine at the final concentration of 600 μg/mL for 6 h and then treated with or without NIR irradiation as describe above. After a 24 h incubation, cells were harvested and stained via the Annexin V-FITC/PI apoptosis detection kit according to the manufacturer’s instructions, followed by flow cytometric analysis.

**Morphology observation of Raji cells after treatment with photonanomedicine**

Briefly, Raji cells were seeded in a 6-well plate at a density of 5×10^5^ cells per well and allowed to cultivate overnight. Thereafter, cells were treated with PpIX-lacking photonanomedicne, CA-lacking photonanomedicne or photonanomedicine at the final concentration of 600 μg/mL for 6 h and applied with or without NIR irradiation. The cells were then incubated at 37 °C for another 24 h. Finally, the cells were washed and collected for scanning electron microscopy (SEM) observation.

**CD20 receptors clustering induced by photonanomedicne**

Briefly, Raji cells were seeded in a 12-well plate at a density of 1×10^5^ cells per well and cultured for 24 h. After that, the cells were incubated with FITC-labeled anti-CD20 antibody (400 nM) and photonanomedicine (600 μg/mL) for another 6 h. Afterwards, the cells were irradiated with a 980 nm NIR laser (2 W/cm^2^, 5 min break after 10 min of irradiation) for 30 min. Finally, the cells were washed and stained for the observing via confocal laser scanning microscopy. The cells without NIR irradiation was used as a control group.

**Intracellular ROS detection**

In order to detect ROS in cells, Raji cells were cultured into 12-well plate and incubated overnight. After that, PpIX-lacking photonanomedicne, CA-lacking photonanomedicne or photonanomedicine (600 μg/mL) was incubated with cells for 6 h and then irradiated with or without NIR laser (2 W/cm^2^, 5 min break after 10 min of irradiation) for different time periods. Finally, DCFH-DA solution (20 μM) was added to culture medium for additional 20 min. The fluorescence intensity of DCF in cells was detected via flow cytometry, as well as examined by confocal microscopy.

**Calcium influx investigation**

In a typical experiment, 5×10^5^ Raji cells were seeded in a 6-well plate and allowed to cultivate overnight. Then the cells were exposed to PpIX-lacking photonanomedicne, CA-lacking photonanomedicne or photonanomedicine at the final concentration of 600 μg/mL. After co-incubation for 6 h, the cells were irradiate with or without NIR laser for 30 min. Subsequently, Fluo-4 AM (5×10^-6^ M), which is an intracellular calcium indicator was added and allowed to culture for another 30 min at 37 °C. Finally, the cells were observed by fluorescence microscopy or analyzed via flow cytometry.

**Mitochondrial membrane potential study**

The mitochondria membrane potential (△ψm) were measured with mitochondria membrane potential assay kit, which is named JC-1 probe. Briefly, Raji cells were seeded at a density of 1×10^5^ cells per well in 12-well plate and allowed to incubate overnight. After that, PpIX-lacking photonanomedicne, CA-lacking photonanomedicne or photonanomedicine (at the final concentration of 600 μg/mL) was added and co-cultivate for another 6 h. Then, the cells were irradiated with or without a 980 nm NIR laser at 2 W/cm^2^ for 30 min. Next, the cells were washed with PBS and processed with JC-1 probe according to the product description. Finally, the cells were observed by fluorescence microscopy or analyzed via flow cytometry.

**Apoptotic proteins detection**

For western blot, Raji cells were suspended in serum-free medium and seeded in 6-well plate at a density of 5×10^5^ cells per well and allowed to cultivate overnight. The cells were exposed to PpIX-lacking photonanomedicne, CA-lacking photonanomedicne or photonanomedicine (at the final concentration of 600 μg/mL) for 6 h. Afterwards, the cells were treated with or without 980 nm NIR irradiation for 30 min. Then cells were lysed and subjected to SDS-PAGE, and immunoblotted with anti-apoptosis antibodies (Bcl-2 and Bcl-xL) or pro-apoptosis antibodies (Bax, Bak and Caspase-3) according to the manufactory’s protocol. The β-actin was used as the reference. Finally, the proteins were visualized via Chemiluminescence imager (ChemiDoc XRS+, Bio-Rad, USA).

**Cytochrome c release measurement**

Western blot was employed to determine the cytochrome c expression levels changes in mitochondria and cytoplasm of Raji cells. In a typical experiment, Raji cells were inoculated into a 6-well plate with a cell density of 5×10^5^ per cell. After that, PpIX-lacking photonanomedicne, CA-lacking photonanomedicne or photonanomedicine at a final concentration of 600 μg/mL was added and co-incubation for 6 h. The cells were then treated with or without 980 nm NIR irradiation for 30 min. Subsequently, the cells were harvested and cytochrome c from mitochondria and cytoplasm was extracted according to the manufactory’s instruction. Next, the obtained protein samples were immunoblotted with the antibodies of cytochrome c, following the protocol mentioned above. Finally, the proteins were washed and imaged by Chemiluminescence imager.

**Biodistribution of photonanomedicine in Raji tumor bearing mice**

The xenografted tumor models were first established by subcutaneous injection of Raji cells (2×10^7^) dispersed in serum-free RPMI-1640 medium into the right flanks of Balb/c nude mice. Treatment began when the tumor volume reached about 300 mm^3^. Two hundred microliters of Apt-lacking photonanomedicine or photonanomedicine were injected into the mice (three mice per group) via the tail vain and at 2, 6, 12, 24, 48 and 72 h after injection, the treated mice were then anesthetized and the fluorescence images were acquired by Maestro in vivo imaging system. At 72 h post-i.v. injection, the mice were sacrificed to harvest the main tissues (heart, liver, spleen, lung, kidney and tumor). Finally, the fluorescence images were obtained, and the fluorescence intensity of the tissues was measured ex vivo using an in vivo imaging system. In addition, the tumor tissues were sliced and stained for the observation of fluorescence via confocal microscopy.

**In vivo antitumor study**

In total, 2×10^7^ of Raji cells were subcutaneous injected into the right flanks of Balb/c nude mice. When the tumor volume reached about 100 mm^3^, the mice were randomly sorted into six groups (6 mice per group) to respectively receive one of the following treatments once every other day: Saline, NIR, Photonanomedicine, PpIX-lacking photonanomedicine+NIR, CA-lacking photonanomedicine+NIR and photonanomedicine+NIR. 50 mg/kg of the test agents per dose was used in the treatment and at 24 h post-i.v. injection, the 980 nm NIR laser was applied locally for 30 min (2 W/cm^2^, 5 min break after 10 min of irradiation). The body weight and tumor volume were monitored every 2 days and the survival time was also recorded. The tumor volume was calculated using the formula: a^2^×b/2, in which a and b represent the smallest and largest diameters of the corresponding tumor, respectively. At the end of monitoring on day 31, the mice were sacrificed and main tissues (heart, liver, spleen, lung, kidney and tumor) were harvested and fixed in 4% paraformaldehyde, embedded in paraffin, cut into 5 μm slices, and stained with H&E, then examined under a light microscope. The apoptosis of tumor tissues also be studied via immunofluorescence of Ki67, Tunel and Caspase-3 staining.

**Biosafety investigation in vivo**

On day 31 after treatment, possible adverse effects of test agents were assessed via detect the counts of white blood cells (WBC) and platelets (PLT), as well as the levels of creatinine (CRE), blood urea nitrogen (BUN), aspartate transaminase (AST) and alanine aminotransferase (ALT). The above indexes were measured by using an automated Beckman Analyzer (Beckman Instruments GmbH, Munich, Germany). All samples were measured in triplicate.

**Statistical analysis**

All the data were presented as means ± standard deviation (SD). Sample size (n) for each statistical analysis were more than or equal to three (n ≥ 3). Statistical analysis of differences between two groups was evaluated with unpaired Student’s t test. Multiple-group comparisons were performed using one-way ANOVA. P values < 0.05 was considered to indicate statistical significance (*p < 0.05, **p < 0.01 and ***p < 0.001). GraphPad Prism 8 software was used for all the statistical analyses.

**
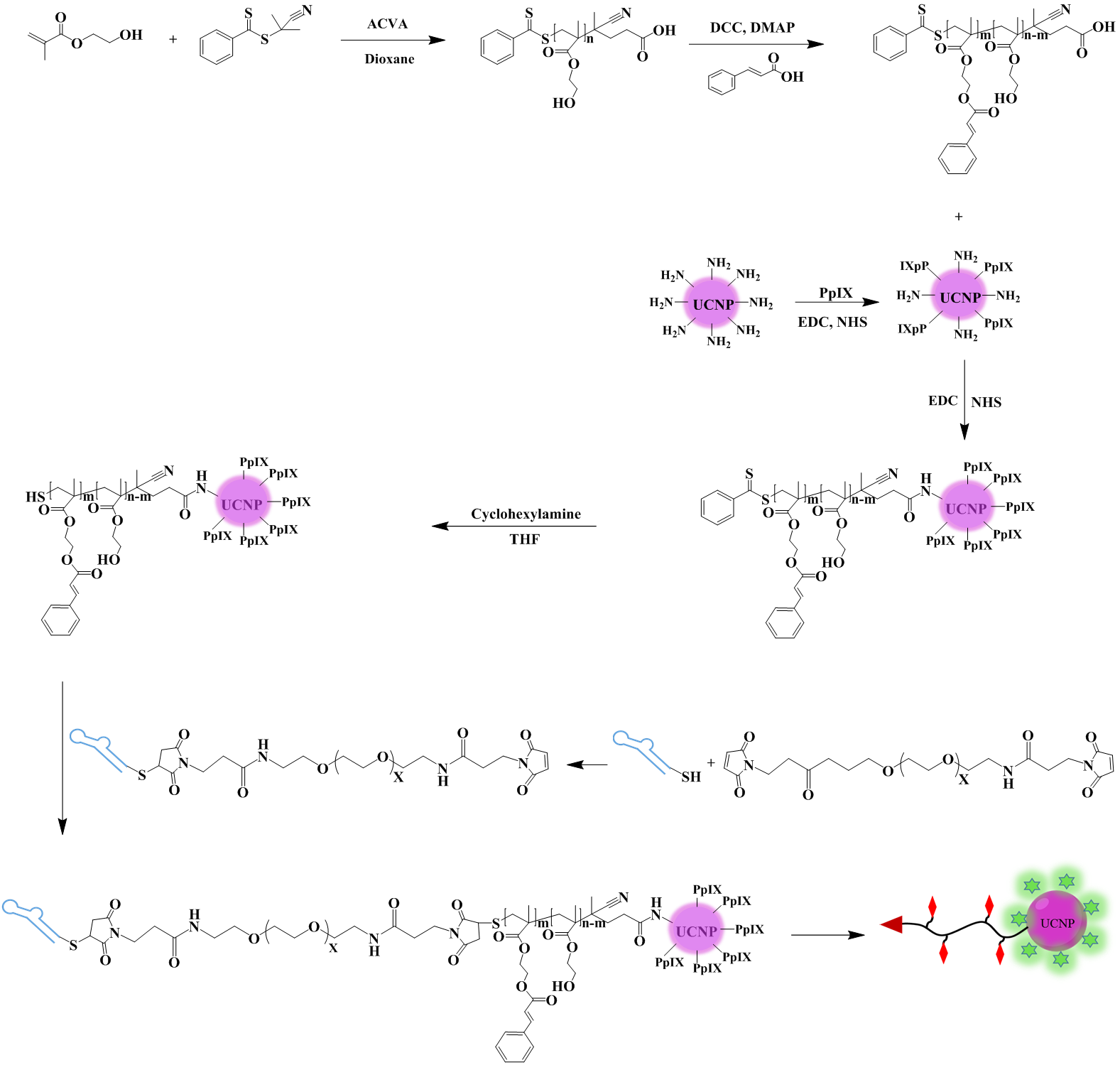
**

**Scheme S1.** Illustration of the synthesize procedure of photonanomedicine.


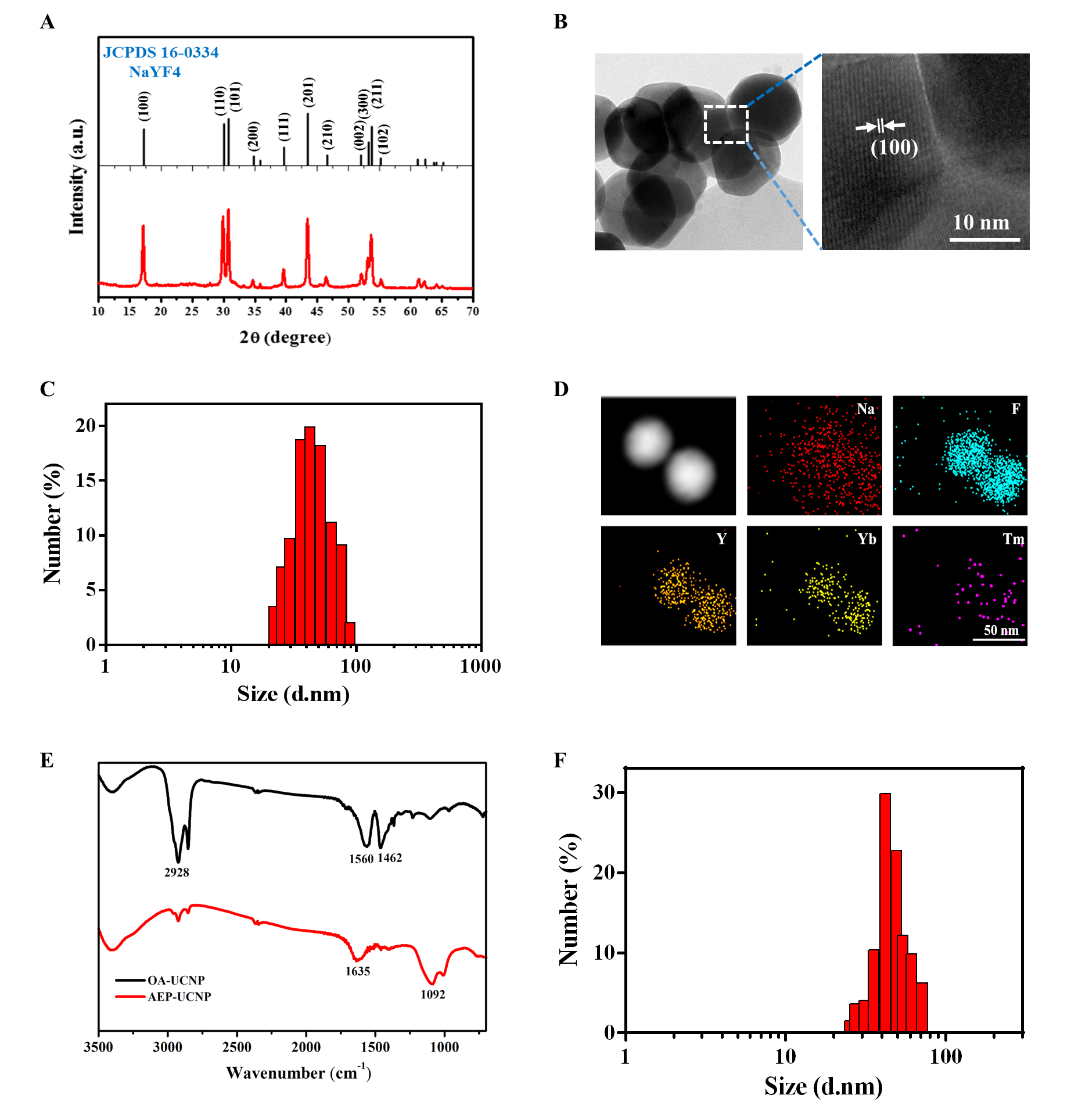


**Fig. S1.** Characteristic of UCNP and PpIX-modified UCNP. (A) X-ray diffraction pattern of UCNP. (B) High resolution transmission electron microscopy (HR-TEM) image of UCNP. (C) The size distribution of UCNP. (D) An energy dispersion spectrum (EDS)-TEM photograph of UCNP. (E) Fourier transform infrared spectroscopy (FTIR) absorption of OA-stabilized UCNP (black curve) and amino-functionalized UCNP (red curve). (F) The size distribution of PpIX modified UCNP.

**
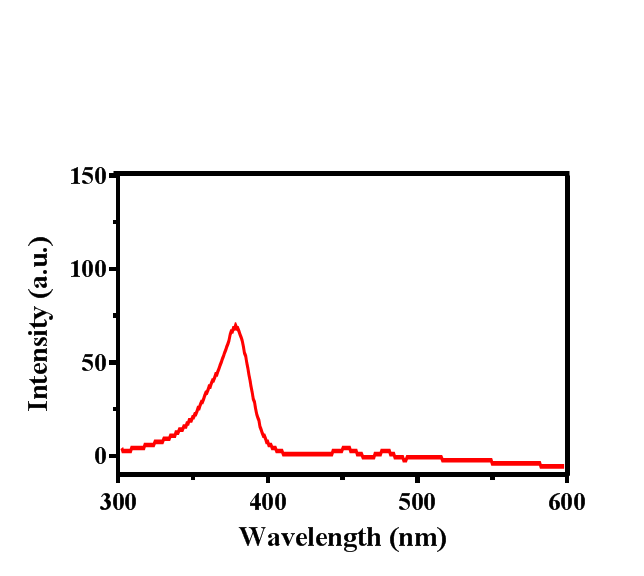
**

**Fig. S2.** The absorption spectrum of the PpIX.


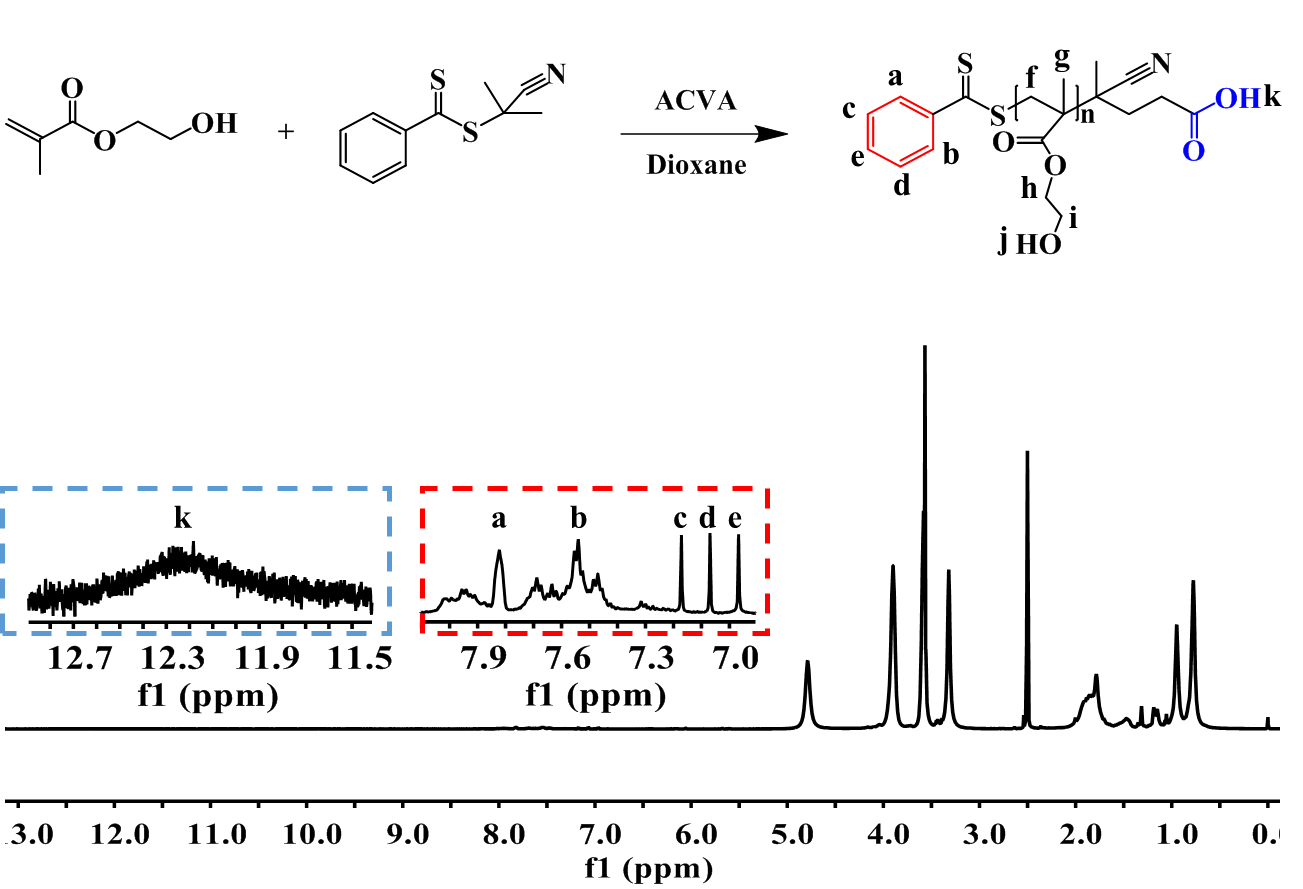


**Fig. S3.** Synthetic and ^1^H-NMR spectrum of the polymer. The square frame marked the specific proton peaks of the polymer.


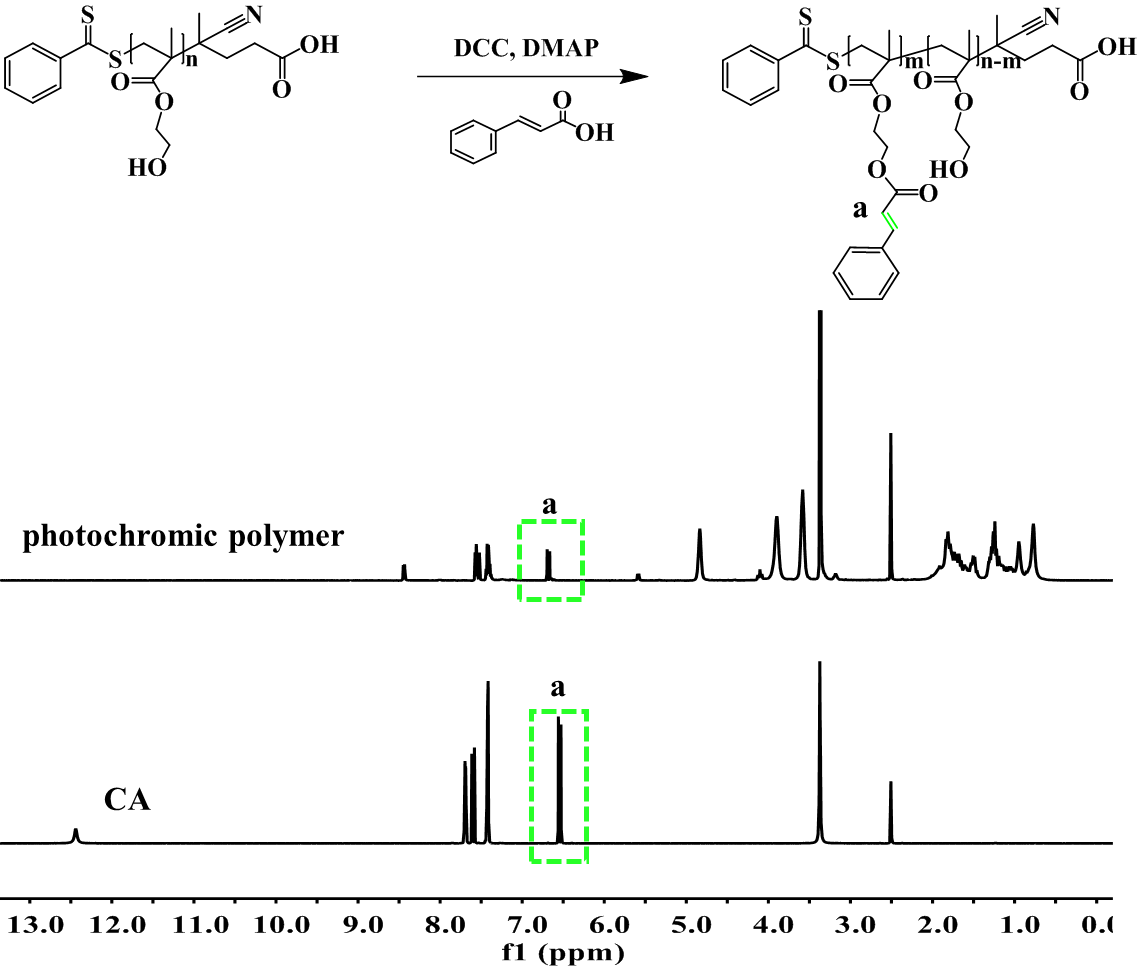


**Fig. S4.** Synthetic and ^1^H-NMR spectrum of the photochromic polymer. The green square frame marked the specific proton peaks of the photochromic polymer.


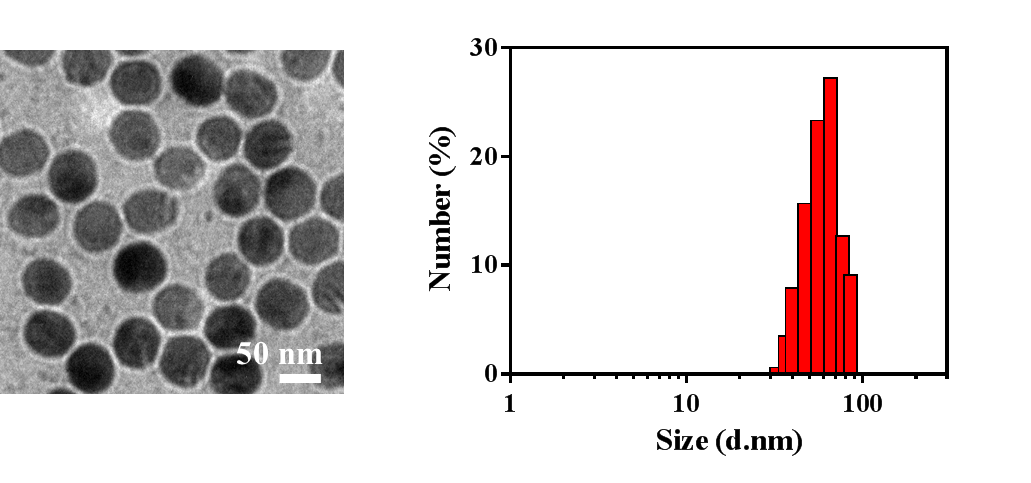


**Fig. S5.** A representative TEM photograph and size distribution of Apt-lacking photonanomedicine.


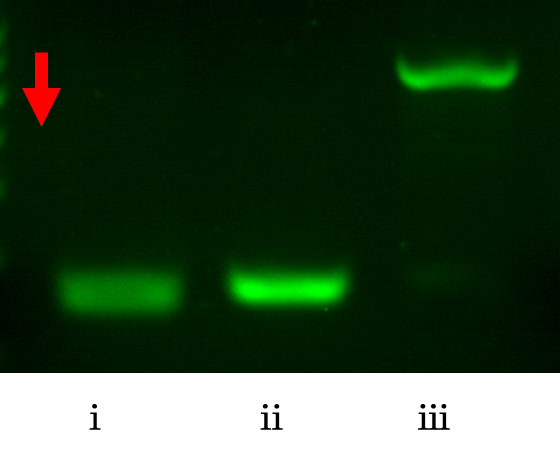


**Fig. S6.** A representative image of agarose gel electrophoresis for FITC tagged Apt-lacking photonanomedicine (i), mixture of Apt-lacking photonanomedicine and aptamer (ii), and photonanomedicine (iii).


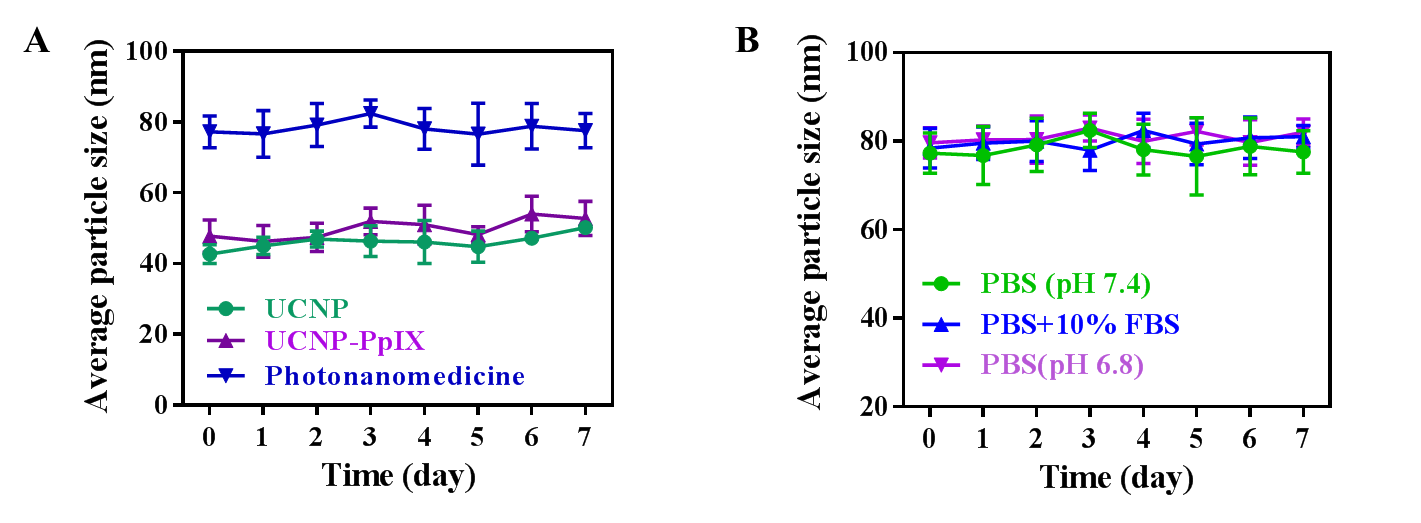


**Fig. S7.** (A) The stability of UCNP, UCNP-PpIX and photonanomedicine nanoparticles in PBS for various time periods. (B)The stability of photonanomedicine in PBS, PBS containing 10% FBS and acid PBS (pH 6.8) for various time periods.

**Fig. S8.** Cell viability of HUVEC, HEK293 and NIH3T3 cells after incubation with photonanomedicine at various concentrations for 48 h.

**Fig. S9.** Cell viability of Raji cells after irradiation with 980 nm NIR laser (2 W/cm^2^, 5 min break after 10 min of irradiation) under different irradiation time.


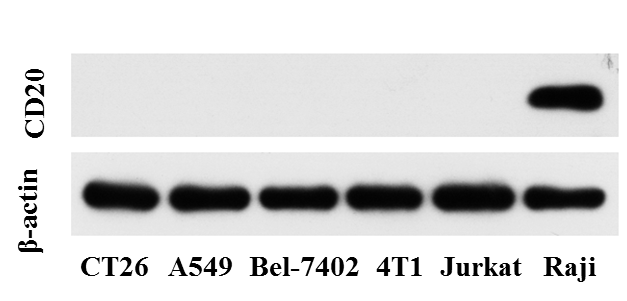


**Fig. S10.** CD20 expression levels of various cancer cell lines.


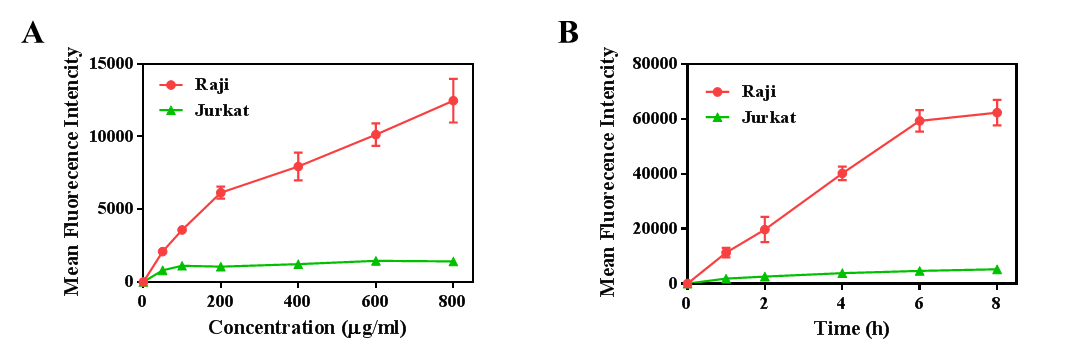


**Fig. S11.** Flow cytometry analysis of Raji and Jurkat cells incubated with photonanomedicine at different concentrations (A) and time points (B).


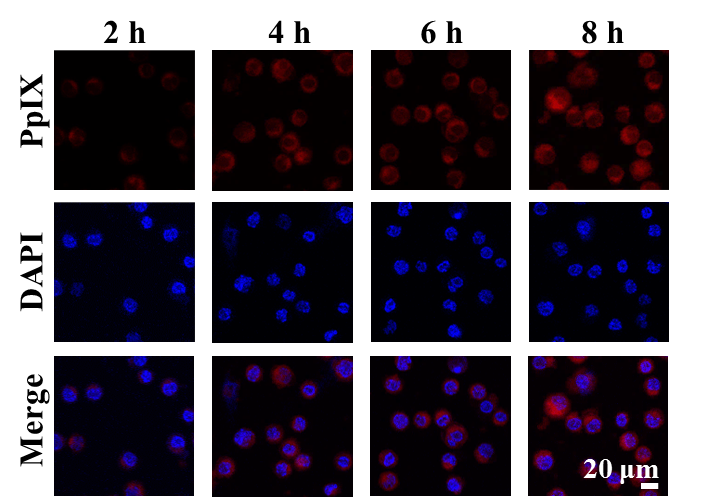


**Fig. S12.** Confocal microscopy images of Jurkat cells after exposure to photonanomedicine for various time periods.


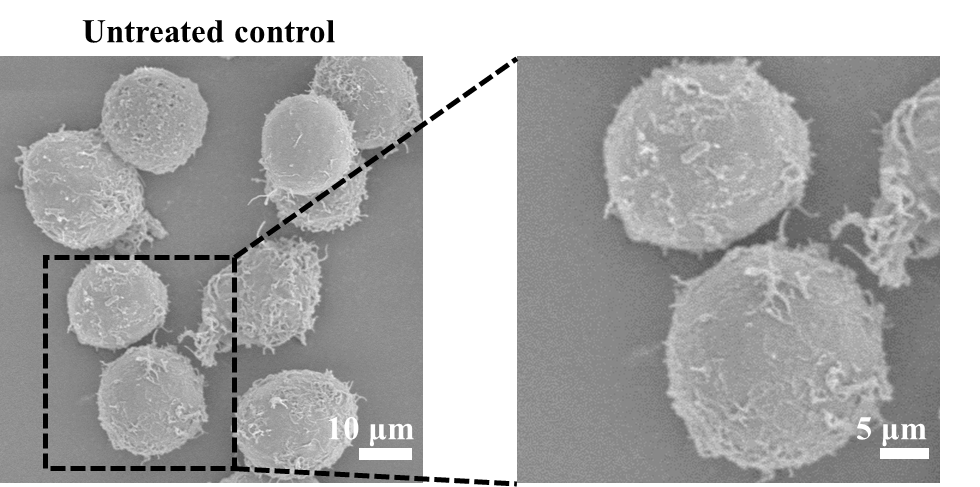


**Fig. S13.** SEM images of Raji cells without crosslinking of photonanomedicine at cell surface.

**Fig. S14.** The mean fluorescence intensity of DCF was quantitatively calculated by Image J based on Fig. 3B. Data were presented as means ± SD, ns means no significant difference, **p < 0.01.


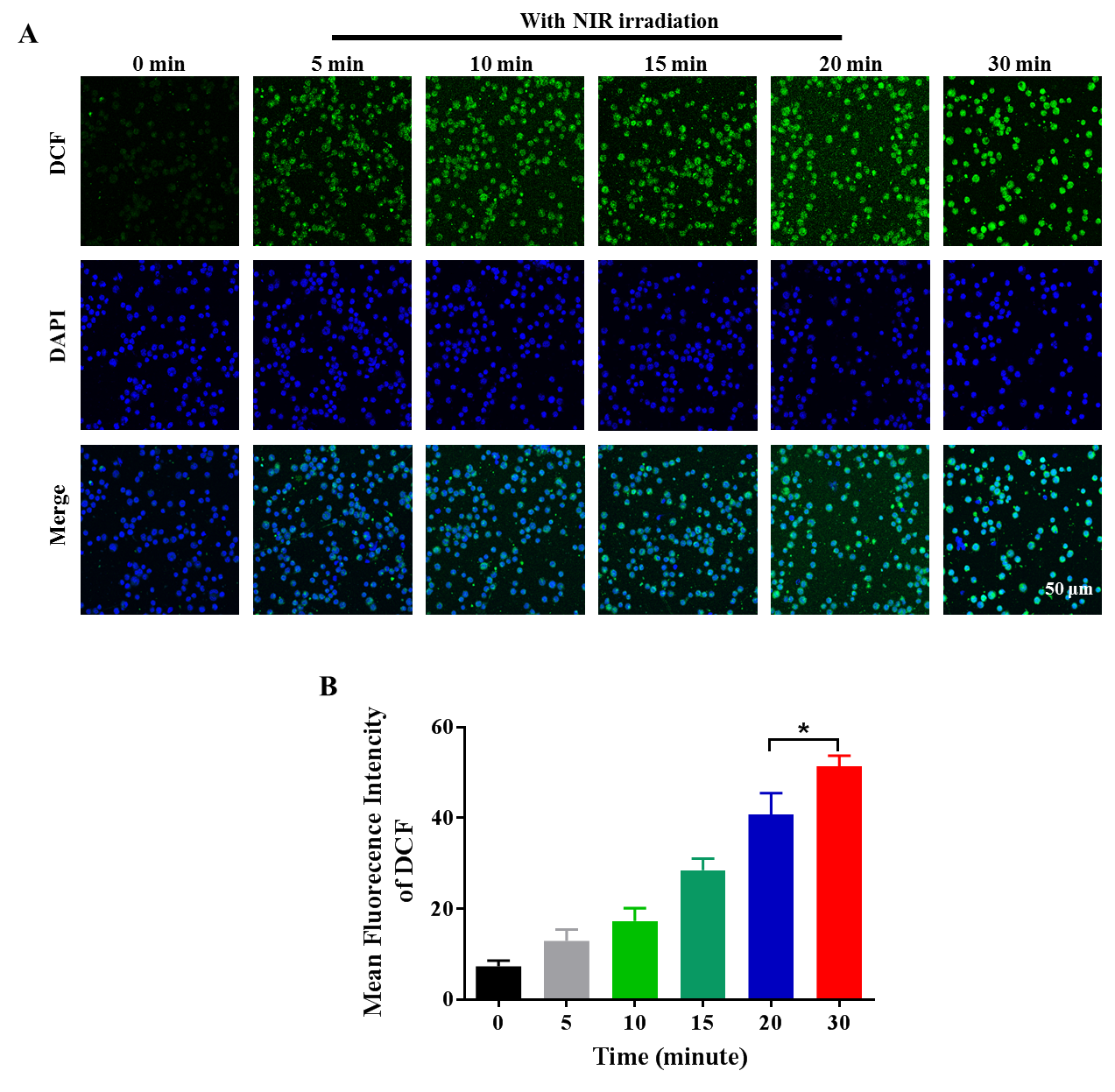


**Fig. S15.** Intracellular ROS generation in Raji cells was measured via confocal microscopy after treatment with photonanomedicine for various time periods. Data were presented as means ± SD, *p < 0.05.

**Fig. S16.** The mean fluorescence intensity of Fluo-4 AM was quantitatively calculated by Image J based on Fig. 3E. Data were presented as means ± SD, *p < 0.05, ***p < 0.001.


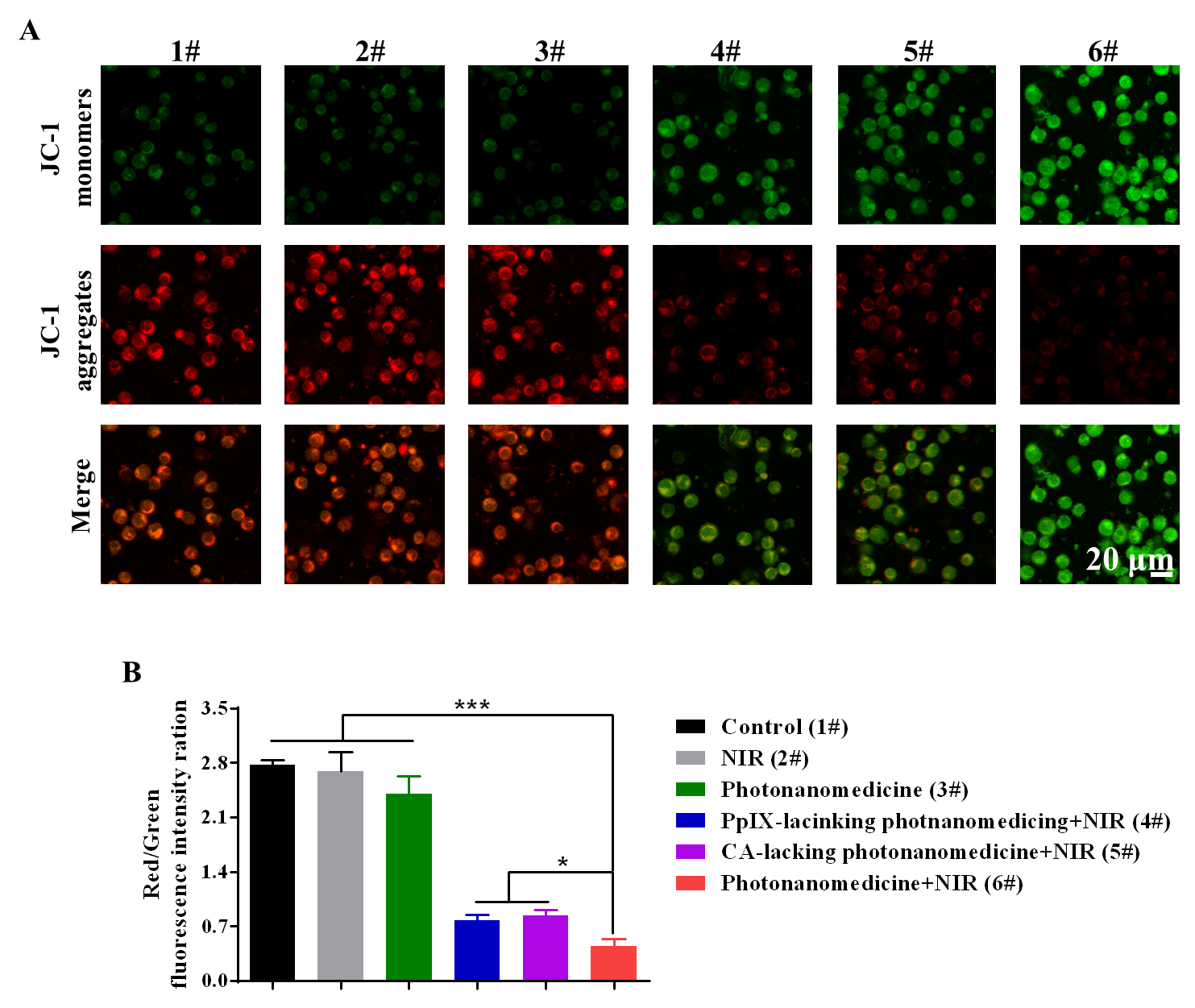


**Fig. S17.** Fluorescence images of Raji cells stained with JC-1 after various treatments (A) and the red/green fluorescence intensity ration of JC-1was analyzed via Image J (B). Data were presented as means ± SD, *p < 0.05, ***p < 0.001.

**Fig. S18.** Quantitatively analysis of relative protein expression of Bcl-2, Bcl-xL, Bax, Bak and Caspase-3 in Raji cells after various treatments based on Fig. 3H. Data were presented as means ± SD, **p < 0.01, ***p < 0.001.

**Fig. S19.** Quantitatively analysis of relative Cyt c protein expression in Raji cells after various treatments based on Fig. 3I and J. Data were presented as means ± SD, *p < 0.05.


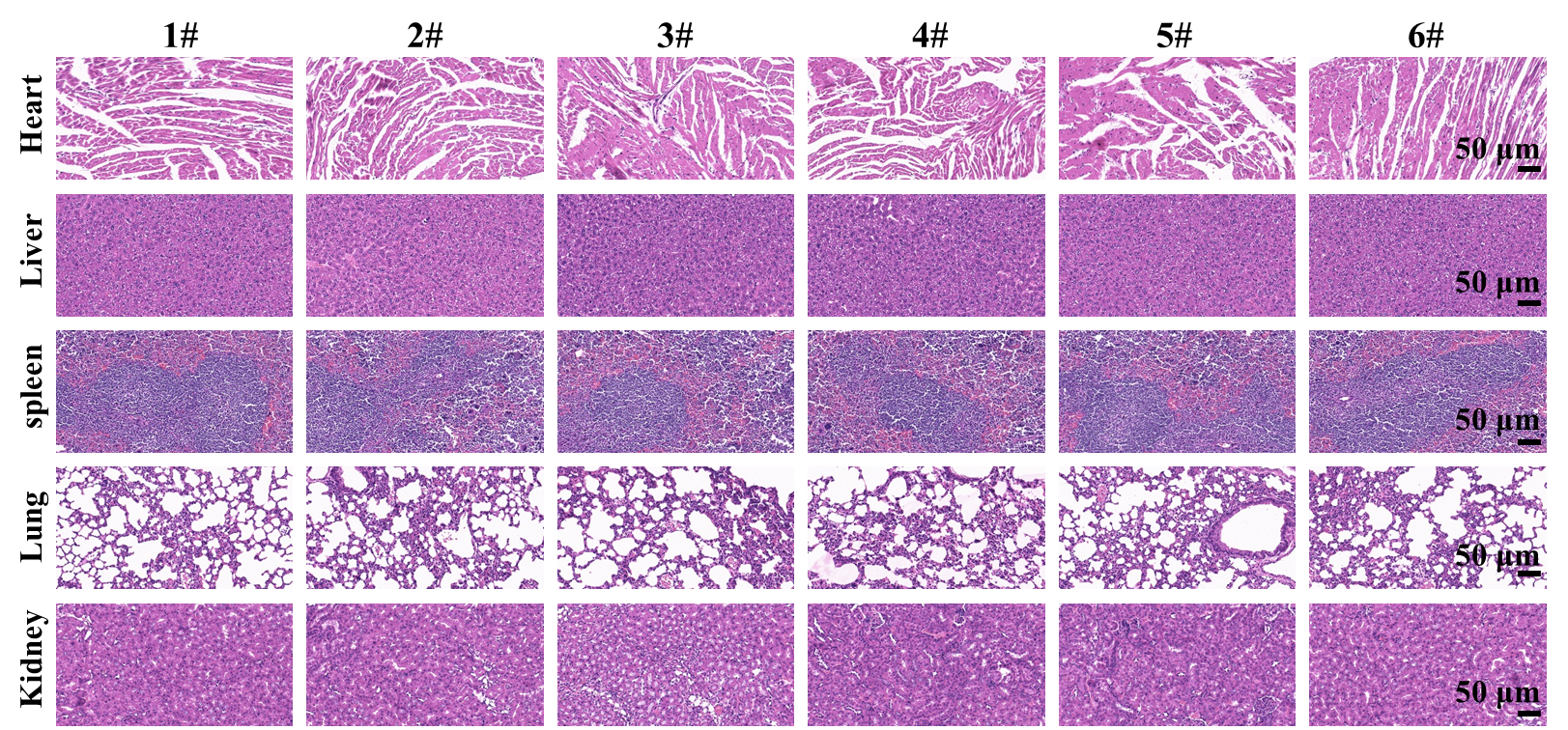


**Fig. S20.** Confocal microscopy images of H&E stained major organs (heart, liver, spleen, lung and kidney) of mice with various treatments via intravenous injection at the end of observation. 1#: Saline; 2#: NIR; 3#: Photonanomedicine; 4#: PpIX-lacking photnanomedicine+NIR; 5#: CA-lacking photnanomedicine+NIR; 6#: Photonanomedicine+NIR.
